# Oral pathogen aggravates atherosclerosis by inducing smooth muscle cell apoptosis and repressing macrophage efferocytosis

**DOI:** 10.1038/s41368-023-00232-5

**Published:** 2023-06-28

**Authors:** Hanyu Xie, Ziyue Qin, Ziji Ling, Xiao Ge, Hang Zhang, Shuyu Guo, Laikui Liu, Kai Zheng, Hongbing Jiang, Rongyao Xu

**Affiliations:** 1 Department of Oral and Maxillofacial Surgery, Affiliated Hospital of Stomatology, Nanjing Medical University, Nanjing, China; 2 Jiangsu Key Laboratory of Oral Diseases, Nanjing Medical University, Nanjing, China; 3 Jiangsu Province Engineering Research Center of Stomatological Translational Medicine, Nanjing Medical University, Nanjing, China; 4 Department of Periodontology, Affiliated Hospital of Stomatology, Nanjing Medical University, Nanjing, China; 5 Department of Orthodontics, Affiliated Hospital of Stomatology, Nanjing Medical University, Nanjing, China

**Keywords:** Periodontitis, Aortic diseases

## Abstract

Periodontitis imparting the increased risk of atherosclerotic cardiovascular diseases is partially due to the immune subversion of the oral pathogen, particularly the *Porphyromonas gingivalis* (*P. gingivalis*), by inducing apoptosis. However, it remains obscure whether accumulated apoptotic cells in *P. gingivalis*-accelerated plaque formation are associated with impaired macrophage clearance. Here, we show that smooth muscle cells (SMCs) have a greater susceptibility to *P. gingivalis*-induced apoptosis than endothelial cells through TLR2 pathway activation. Meanwhile, large amounts of miR-143/145 in *P.gingivalis*-infected SMCs are extracellularly released and captured by macrophages. Then, these miR-143/145 are translocated into the nucleus to promote Siglec-G transcription, which represses macrophage efferocytosis. By constructing three genetic mouse models, we further confirm the in vivo roles of TLR2 and miR-143/145 in *P. gingivalis*-accelerated atherosclerosis. Therapeutically, we develop *P.gingivalis*-pretreated macrophage membranes to coat metronidazole and anti-Siglec-G antibodies for treating atherosclerosis and periodontitis simultaneously. Our findings extend the knowledge of the mechanism and therapeutic strategy in oral pathogen-associated systemic diseases.

## Introduction

Periodontitis is a dysbiotic inflammatory disease associated with oral pathogen subversion of the host immune response that disrupts tooth-supporting tissues and ultimately leads to tooth loss. Microbial immune subversion not only perturbs periodontal tissue homeostasis at the oral site but also compromises immune surveillance and homeostasis at systemic sites, such as atherosclerotic plaques, placenta, and brain, which in turn promotes or accelerates pathogenic processes.^1–3^ As a model pathogen for investigating microbial immune subversion,^4^ *P. gingivalis* is associated with atherosclerotic cardiovascular disease, as shown by clinical and in vitro studies confirming the atherogenic potential of *P. gingivalis* in atherosclerosis.^5^ The translocation of *P. gingivalis* into systemic circulation occurs when the gingival ulceration or debridement in periodontal pockets.^6,7^ Then, circulating *P. gingivalis* contributes to aortic immune subversion by hijacking macrophages and/or dendritic cells into inflammatory vascular sites,^8,9^ and these colonized *P. gingivalis* can induce cellular apoptosis and stimulate inflammatory cytokine production by activating the Toll-like receptor 2 (TLR2) pathway.^8,10–12^ However, little is known about whether the TLR2 pathway could also disturb the clearance of apoptotic cells in *P. gingivalis*-infected atherosclerotic plaques.

The immune-subversive strategy used by *P. gingivalis* is a common way to evade immune-mediated killing by interacting with host immune responses, including neutrophils or macrophages.^13^ *P. gingivalis* can suppress the phagocytosis of macrophages to enhance its fitness by inhibiting nitric oxide synthase and NLRP3 inflammasome activation.^8,14^ Notably, bacterial infection leads to the dysregulation of immune checkpoints and impairs macrophage efferocytosis (a process to clear apoptotic cells), which results in a chronical inflammatory response.^15,16^ During the development of atherosclerosis, accumulated apoptotic cells promote pathogenic plaque formation due to the activation of immune checkpoints, such as the CD47-SIRPα anti-phagocytic axis.^17^ Another anti-phagocytic signal, sialic acid-binding immunoglobulin-like lectin G (Siglec-10 in humans or Siglec-G in mice) expressed by macrophages, is able to inhibit the phagocytic capacity,^18^ while genetic ablation and antibody blockade of Siglec-10 robustly augments macrophage phagocytosis.^19^ Notably, Siglec-G deficiency in atherosclerosis-prone mice reduces plaque formation by inhibiting the proinflammatory properties of oxidized low-density lipoprotein (OxLDL).^20^ Although these observations have revealed the roles of Siglec-G in promoting atherosclerosis, whether Siglec-G has involved in *P. gingivalis*-accelerated atherosclerosis by regulating macrophage efferocytosis remains elusive.

Vascular smooth muscle cells (SMCs) in atherosclerosis functionally differ from normal tissue, which is transformed from a contractile/nonproliferative phenotype to a migratory/proliferative phenotype. Apart from that, apoptosis of SMCs is especially detrimental to atherosclerotic progression, as accumulated apoptotic SMCs destabilize the fibrous cap and contribute to plaque rupture.^21^ The microRNA-143/145 (miR-143/145) cluster, which is involved in the regulation of SMC phenotype and function, has been reported to be critical in the development of atherosclerosis. However, several studies unveiled downregulated miR-143/145 expression in atherosclerotic arteries,^22,23^ while others detected increased miR-143/145 expression in human and mouse carotid atherosclerotic plaques.^24,25^ In addition, deleting miR-143/145 in atherosclerosis-prone mice reduced plaque size and macrophage infiltration.^25^ Notably, salivary miR-143 has recently been identified as a novel diagnostic biomarker for chronic periodontitis.^26^ Therefore, we hypothesized that the high expression of miR-143/145 in some arteriosclerotic plaques might be associated with oral pathogen infection, leading to accelerated atherosclerosis. Although miR-143/145 is crucial for manipulating SMCs phenotypic switching, its role in *P. gingivalis*-accelerated atherosclerosis needs to be further explored to clarify the observed contradiction of miR-143/145 expression in plaques. In our study, we first aimed to dissect how *P. gingivalis* subverts immune homeostasis to accelerate arteriosclerotic progression and regulates miR-143/145 expression as well as the possible mechanisms involved. Second, based on the strategy of antibacterial intervention and restoring immune clearance, we also developed a promising targeting approach to treat *P. gingivalis*-infected periodontitis and atherosclerosis simultaneously.

## Results

### Increased miR-143/145 levels and apoptotic cells in *P. gingivalis*-infected periodontitis or atherosclerosis

**Fig. 1:**
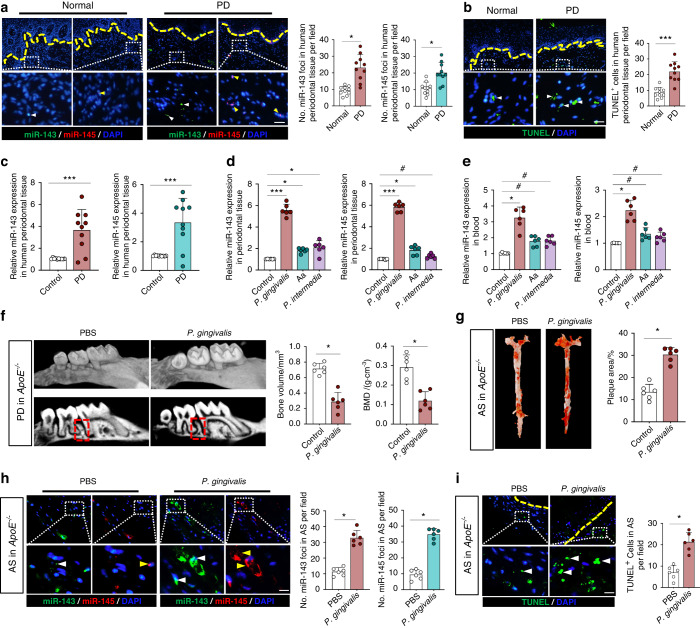
*P. gingivalis*-infected periodontitis or atherosclerosis is associated with increased miR-143/145 levels and apoptotic cells. **a** FISH staining examined the miR-143/145 expression in healthy individuals and periodontitis (PD) patients. White and yellow arrowhead respectively indicates miR-143 and miR-145. The yellow dotted line indicates the basement membrane. *n* = 10. Bar: 50 μm. **b** TUNEL staining showed the difference in apoptotic cells in periodontium. *n* = 10. Bar: 50 μm. **c** Relative miR-143/145 levels in human periodontium were measured by qRT-PCR from the control or periodontitis group. *n* = 10. **d** Expression of miR-143/145 in the periodontium and **e** blood circulation by qRT-PCR from control or *P. gingivalis* o*r A. actinomycetes* or *P. intermedia* treated wild type (WT) mice. *n* = 6. **f** The representative micro-CT images of alveolar bone between the first molar and second molar in *ApoE*^*−/−*^ mice with PBS or *P. gingivalis* exposure after 12 weeks of HFD feeding. The right panel shows the quantitative data on bone volume and bone mineral density (BMD). The red dotted line indicates the region of interest (ROI). PD periodontitis. **g** The representative images of Oil Red O staining atherosclerotic lesions of the aorta en face, with quantitative data at right. *n* = 6. AS atherosclerosis. **h** FISH staining showing the miR-143 (white arrowhead) and miR-145 (yellow arrowhead) expression, and **i** TUNEL staining showing the apoptotic cells in atherosclerotic plaque of *ApoE*^*−/−*^ mice, respectively. *n* = 6. Scale bars: 20 μm. The right panel shows the quantitative data. Results are presented as the mean ± S.D. by one-way ANOVA followed by Tukey multiple comparisons tests or unpaired 2-tailed Student *t*-tests. *P < 0.05; ***P < 0.001; #*P* > 0.05

We first examined miR-143/145 expression in the periodontium from ten periodontitis patients and observed high levels of miR-143/145 in periodontitis patients compared with healthy individuals (Fig. 1a). We also observed more TUNEL-positive cells in periodontitis patients (Fig. 1b), indicating the ability of an oral pathogen to induce apoptosis. Consistently, the increased level of miR-143/145 was further confirmed by qRT-PCR in periodontitis patients compared with the corresponding controls (Fig. 1c). Given that periodontitis-mediated atherosclerotic progression is associated with the invasion of oral bacteria, the most frequently detected bacteria in plaques of atherosclerotic patients including *Porphyromonas gingivalis* (*P. gingivalis*)*, Actinobacillus actinomycetemcomitans* (*A. actinomycetes*) and *Prevotella intermedia* (*P. intermedia*)^1,27^ were subjected to the infection of wild type (WT) mice via oral inoculation for 4 weeks. Interestingly, in *P. gingivalis-*infected WT mice, miR-143/145 showed the most significant increase in both periodontal tissue and blood circulation compared with Aa- or Pi-infected mice (Fig. 1d, e). Besides, we also detected the miR-143/145 expression in aortic root tissue but found no significant alternation with the *P.gingivalis* infection (Fig. S1a). Next, to confirm the effect of *P. gingivalis* infection on the development of atherosclerosis, *ApoE*^*−/−*^ mice were orally infected with *P. gingivalis* for 12 weeks. Compared to the mice without *P. gingivalis* infection, we observed a significant reduction in bone volume and bone mineral density (BMD) in the region between the first and second molars after *P. gingivalis* infection (Fig. 1f). Meanwhile, the number of TRAP-positive osteoclasts was higher in *P. gingivalis*-infected *ApoE*^*−/−*^ mice than that in the control group (Fig. S1b). After confirming the residence of *P. gingivalis* in atherosclerotic plaques (Fig. S1c), the aortic tree and root were subjected to Oil Red O staining, showing increased plaque area in *P. gingivalis*-infected *ApoE*^*−/−*^ mice (Figs. 1g and S1d). In addition, to detect the role of *P. gingivalis* in regulating miR-143/145 expression and cellular apoptosis, we collected periodontal tissue and atherosclerotic plaques from *P. gingivalis*-infected *ApoE*^*−/−*^ mice and observed increased miR-143/145 expression and apoptotic cells in both tissues (Figs. 1h, i and S1e, f). These results suggest that *P. gingivalis* infection accelerates atherosclerotic development accompanied by highly expressed miR-143/145 and increased apoptotic cells.

### *P. gingivalis* induces SMCs apoptosis and in turn contributes to miR-143/145 extracellular releasing

**Fig. 2:**
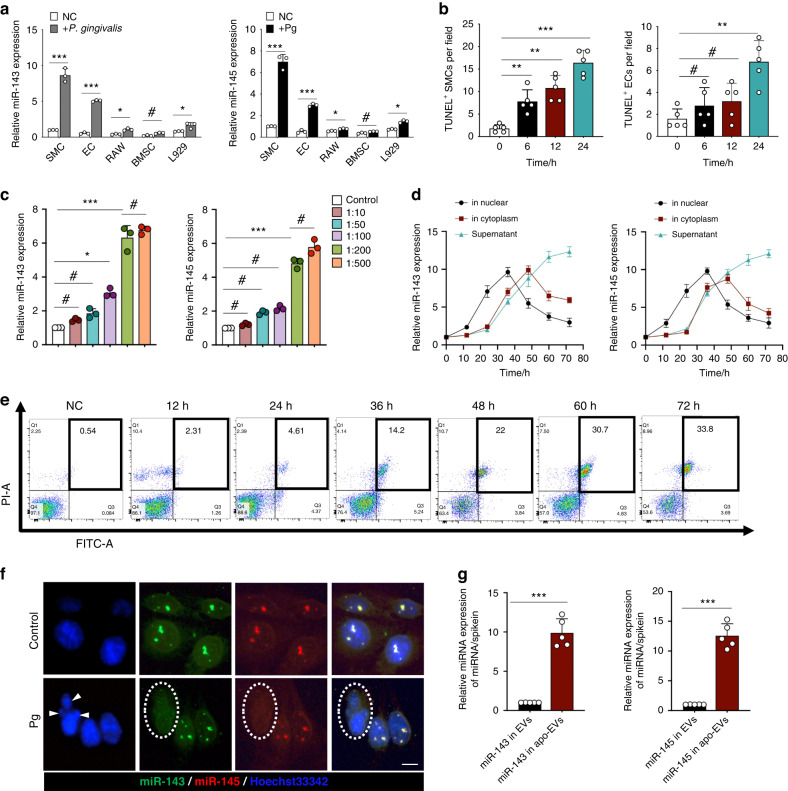
*P. gingivalis* promotes SMCs apoptosis and extracellularly releases miR-143/145 via apoEVs. **a** With the exposure of PBS or *P. gingivalis*, relative miR-143/145 levels in mouse ECs, SMCs, L929, or BMSCs were measured by qRT-PCR. **b** Quantitative analysis of TUNEL-positive SMCs at different times of *P. gingivali*s stimulation. *n* = 5. **c** Relative miR-143/145 expression in SMCs treated with different *P. gingivalis* concentrations were measured by qRT-PCR. **d** Relative miR-143/145 expression in nucleus, cytoplasm, and supernatant of SMCs with *P. gingivalis* stimulation at different times by qRT-PCR. **e** Flow cytometry revealed the numbers of apoptotic cells when the *P. gingivalis* infected SMCs at different times. **f** Nuclear and cytoplasmic distribution of miR-143 and miR-145 in PBS- and *P. gingivalis*-treated SMCs by FISH assay. Scale bars: 20 μm. A white arrowhead indicates a cracked nucleus. The White dotted line indicates the apoptotic SMCs. **g** miR-143 and miR-145 loaded in apoEVs from PBS- and *P. gingivali*s-treated SMCs were examined by qRT-PCR. Results are presented as the mean ± S.D. by one-way ANOVA followed by Tukey multiple comparisons tests or unpaired 2-tailed Student *t*-tests. *P < 0.05; **P < 0.01, ***P < 0.001; #*P* > 0.05

Next, to dissect which cell primarily releases miR-143/145 during *P. gingivalis* infection, we selected candidate cells that exist in the aortic microenvironment, including endothelial cells (ECs, constituent cells of intima), SMCs (constituent cells of media), L929 cells (fibroblast cells, constituent cells of adventitia), RAW264.7 cells (macrophages that modulate innate immunity) and bone marrow mesenchymal stem cells (BMSCs, characterized by multidirectional differentiation), and co-cultured them with *P. gingivalis* for 24 h. SMCs not only had the basally high expression of miR-143/145 but also presented the most significant upregulation of miR-143/145 in the presence of *P. gingivalis* infection compared with that in ECs, L929, RAW264.7, and BMSCs (Fig. 2a). We also noticed the relatively elevated miR-143/145 in ECs with *P. gingivalis* infection. Since ECs-derived miR-143/145 can also control SMCs phenotypes and atherosclerotic lesion formation,^28^ we then compared the influence of *P. gingivalis* on apoptotic induction between SMCs and ECs. As a consequence, the number of dead SMCs and ECs both increased in a *P. gingivalis* MOI-dependent manner, but interestingly, almost all of the SMCs were dead at an MOI of 200 for 24 h, while numerous ECs were still alive in response to an MOI of 500 (Fig. S2a). Furthermore, significant SMCs apoptosis occurred after 6 h of *P. gingivalis* stimulation, as demonstrated by TUNEL staining, but a few apoptotic ECs appeared within 12 h of stimulation (Figs. 2b and S2b). In addition, the number of apoptotic SMCs was also higher in the atherosclerotic plaques with *P. gingivalis* infection in *ApoE*^*−/−*^ mice as compared with the counterparts (Fig. S2c). These data suggest that SMCs are not only more sensitive to *P. gingivalis*-induced apoptosis but also play a more important role in *P. gingivalis*-accelerated atherosclerosis than ECs, thus rendering our research predominantly focused on SMCs. We first selected the proper *P. gingivalis* concentration at an MOI of 200, which showed the most pronounced change in miR-143/145 expression (Fig. 2c). To further clarify the dynamic distribution of miR-143/145 in SMCs by *P. gingivalis* infection, the supernatant, cytosolic and nuclear fractions of SMCs were collected for qRT-PCR analysis at different times (Fig. 2d). In the nucleus, miR-143/145 had a relatively rapid rise in response to *P. gingivalis* infection, but an earlier decline at 36 h as compared with that in the supernatant and cytosolic fractions. At 48 h, the decreased miR-143/145 expression in the cytosolic fraction appeared later than that in the nuclear fraction. Strikingly, the miR-143/145 expression in the supernatant continuously increased with prolonged stimulation with *P. gingivalis*. We further detected the apoptotic state of SMCs by flow cytometry at the corresponding time, showing an obvious upregulation of apoptosis at 36 h (Fig. 2e). The above results prompted us to explore the influence of *P. gingivalis*-induced apoptosis on regulating the extracellular secretion of miR-143/145. We first observed the subcellular localization of miR-143/145 in normal SMCs and found significant nuclear aggregation and co-localization of miR-143/145 in SMCs. However, in *P. gingivalis*-induced apoptotic SMCs, miR-143/145 aggregation or co-localization disappeared in nuclear fragmentation (Fig. 2f). This suggested that the previously high aggregation of nuclear miR-143/145 might be largely released into the cytoplasm and extracellular supernatant. In other words, the continuously elevated miR-143/145 in the supernatant was mainly released from SMCs nuclei after *P. gingivalis*-induced apoptosis. Considering the early occurrence of SMCs apoptosis, miR-143/145 in the extracellular space might be secreted by apoptotic cell-derived extracellular vesicles (apoEVs). Thus, apoEVs from *P. gingivalis*-infected SMCs were isolated and characterized by transmission electron microscopy (TEM) (Fig. S3a), size distribution (Fig. S3b), positive TUNEL staining (Fig. S3c) and relevant protein markers (Fig. S3d). Remarkably, we confirmed that miR-143/145 was highly expressed in apoEVs from *P. gingivalis*-stimulated SMCs compared with EVs from unstimulated SMCs (Fig. 2g). We next examined whether the release of miR-143/145 from the nucleus was dependent on caspase activity and found that although the nucleus was ruptured by *P. gingivalis*-induced apoptosis, miR-143/145 were still existed in the nucleus after treatment with the caspase inhibitor zVAD(Ome)fmk (Fig. S3e). Meanwhile, the expression of miR-143/145 in apoEVs was reduced with the treatment of a caspase inhibitor (Fig. S3f), suggesting that exporting miR-143/145 from the nucleus is caspase-dependent. In summary, *P. gingivalis* is more inclined to induce SMCs apoptosis than ECs apoptosis and leads to increased expression of miR-143/145, which is extracellularly released via loaded into apoEVs.

### *P. gingivalis* activates TLR2-NFκB signaling to promote apoptosis and miR-143/145 expression

**Fig. 3:**
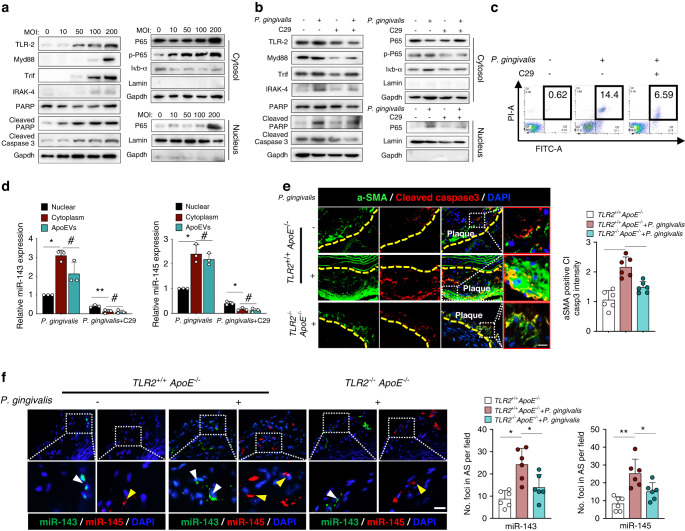
Inhibition of TLR2 signaling alleviates *P. gingivalis*-induced SMCs apoptosis and reduces miR-143/145 expression. **a** Influence of different concentrations of *P. gingivalis* on TLR-2 signaling (MYD88, Trif, and IRAK-4 protein expression), apoptosis-related markers (PARP, cleaved PARP, and Cleaved caspase3 protein expression), and NF-κB pathway (p-P65, P65, and Ikb-α in cytoplasmic extracts, and P65 in nuclear extracts)*.* **b** Influence of inhibiting TLR2 signaling on apoptosis-related markers and NF-κB pathway. **c** Flow cytometry analysis for apoptotic SMCs with the stimulation of *P. gingivalis* and C29 (a TLR2 Inhibitor). **d** Relative miR-143/145 expression in the nucleus, cytoplasm, and apoEVs were measured by qRT-PCR when Pg-infected SMCs were treated with C29. **e** Immunostaining staining of α-SMA and Cleaved caspase3 in atherosclerotic lesions from *TLR2*^*+/+*^*ApoE*^*−/−*^ or *TLR2*^*−/−*^*ApoE*^*−/−*^ mice with *P. gingivalis* exposure or not. The yellow dotted line indicates the boundary between tunica intima and media. *n* = 6. Scale bars: 50 μm. **f** FISH staining showing the miR-143 (white arrowhead) and miR-145 (yellow arrowhead) expression in atherosclerotic plaque of *TLR2*^*+/+*^*ApoE*^*−/−*^ or *TLR2*^*−/−*^*ApoE*^*−/−*^ mice with *P. gingivalis* exposure or not. *n* = 6. Scale bars: 20 μm. The right panel shows the quantitative data. Results are presented as the mean ± S.D by one-way ANOVA followed by Tukey multiple comparisons tests. *P< 0.05; **P< 0.01; #*P* > 0.05

*P. gingivalis* can directly interact with Toll-like receptor 2 (TLR2) to initiate inflammatory signaling such as the nuclear factor-κB (NF-κB) pathway, which is associated with caspase-dependent apoptosis and atherosclerotic progression.^10,17,29^ Analysis of human early (*n* = 13)- and advanced (*n* = 16)- plaques by RNA-seq showed that TLR2 expression was significantly higher in advanced plaques, suggesting the potential role of TLR2 activation in promoting atherosclerosis (Fig. S4a). We further compared the relationship between TLR2 expression and apoptotic or NF-κB pathway-associated markers in 109 atherosclerotic patients, which revealed a positive correlation with CASPI, CASP3, IRAK1, and IRAK3 (Fig. S4b). To explore whether *P. gingivalis* activates TLR2 to initiate the downstream NF-κB pathway, SMCs were infected with different *P. gingivalis* MOI. As expected, the continuous increase in TLR2 expression occurred in a concentration-dependent manner. Simultaneously, the activated TLR2 pathway indicated by elevated Myd88 and Trif expression led to the enhanced NF-κB pathway and upregulated apoptotic induction (Fig. 3a). In contrast, a TLR2 Inhibitor (C29) failed to boost the expression of IRAK-4, Iκb-α, and p-P65, as well as Cleaved PARP and Cleaved caspase3, suggesting that inhibiting the TLR2 pathway could suppress NF-κB signaling and reduce apoptosis of SMCs (Fig. 3b). Moreover, the inhibitory effect of C29 on *P. gingivalis*-induced apoptosis was further validated by flow cytometry assay (Fig. 3c). Noticeably, TLR2 activation has been reported to promote miR-143 expression in inflammatory condition.^30^ Similarly, in this study, when treated with C29 in *P. gingivalis*-infected SMCs, miR-143/145 was indeed diminished in the nucleus, cytoplasm, and apoEVs (Fig. 3d).

To further explore the in vivo role of TLR2 in inducing SMCs apoptosis and miR-143/145 expression during *P. gingivalis*-accelerated atherosclerosis, we constructed a *P. gingivalis*-infected *TLR2*^*−/−*^*ApoE*^*−/−*^ mouse model (Fig. S4c). Rejuvenated BV/TV and BMD phenotypes were observed in *P. gingivalis*-infected *TLR2*^*−/−*^*ApoE*^*−/−*^ mice compared with *P. gingivalis*-infected *TLR2*^*+/+*^*ApoE*^*−/−*^ mice (Fig. S4d). A similar change occurred in osteoclast numbers (Fig. S4e). Notably, TLR2 deficiency reduced atherosclerotic lesions in the aorta en face (Fig. S4f) and diminished the plaque area of the aortic root (Fig. S4g). Crucially, compared with those in the control group, apoptotic SMCs and miR-143/145 levels were substantially decreased in atherosclerotic lesions of *P. gingivalis*-infected *TLR2*^*−/−*^*ApoE*^*−/−*^ mice (Fig. 3e, f). Collectively, these results suggest that *P. gingivalis-*induced SMCs apoptosis and extracellular release of miR-143/145 are associated with the activation of TLR2 signaling, but the pathological influence of increased miR-143/145 levels in the plaque microenvironment needs to be further elucidated.

### miR-143/145 enhances Siglec-G expression to repress macrophage efferocytosis

**Fig. 4:**
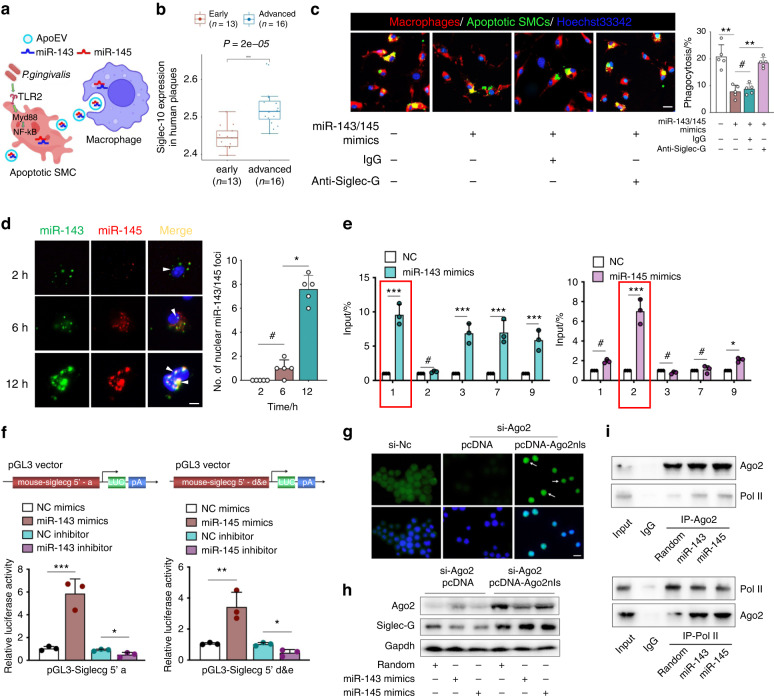
Nuclear miR-143/145 transcriptionally increases Siglec-G expression to repress macrophage efferocytosis. **a** Model of *P. gingivalis*-induced secretion of apoEVs-miR-143/145 captured by macrophages. **b** Analysis of Siglec-10 expression (Siglec-G in mouse) in human early (*n* = 13)- and advanced (*n* = 16)-plaque by RNA-seq from a public database. **c** Efferocytosis ability of macrophages that were treated with miR-143/145 mimics, IgG, or anti-Siglec-G antibodies was detected by phagocytosis assay, with quantitative data at right. Scale bars: 20 μm. **d** Macrophages that were transfected with fluorescently labeled miR-143/145 mimics were observed at different time points, with quantitation of nuclear miR-143/145 foci at right. **e** The relative enrichment of these five putative binding sites of Ago2 in macrophages transfected with negative control (NC) or miR-143/145 mimics. The red dotted line indicates that Site 1 corresponded to miR-143 binding Site a, and Site 2 corresponded to miR-145 binding Sites (**d**) and (**e**). **f** Wild type plasmid based on Site a region of Siglec-G promoter was subjected to dual-luciferase reporter assay in response to NC mimics, miR-143 mimics, NC inhibitor or miR-143 inhibitor. Another wild-type plasmid based on Sites (**d**) and (**e**) region of Siglec-G promoter was subjected to dual-luciferase reporter assay in response to NC mimics, miR-145 mimics, NC inhibitor, or miR-145 inhibitor. **g** Validating the effect of the si-Ago2 on Ago2 expression and nuclear localization of Ago2nls. Scale bars: 10 μm. **h** Western blot analysis shows that miR-143/145 failed to activate Siglec-G expression in the absence of Ago2, but retained Siglec-G expression once with nuclear Ago2 re-expression in Ago2 knockdown macrophages. **i** Western blot showing co-IP between Ago2 and POL II in macrophages with miR-143/145 mimics treatment. Results are presented as the mean ± S.D. by one-way ANOVA followed by Tukey multiple comparisons tests. *P < 0.05; **P < 0.01, ***P < 0.001; #*P* > 0.05

Having documented the presence of large apoEVs-miR-143/145 in the extracellular environment by in vivo and in vitro experiments, we next investigated the subsequent biological processes and functional mechanism of miR-143/145 in *P. gingivalis-*accelerated atherosclerosis. Since apoEVs could be efficiently captured by macrophages (Fig. 4a), SMCs-derived apoEVs labeled with DiO were co-cultured with macrophages, leading to an obvious integration into macrophages (Fig. S5a). Next, we wanted to decipher whether the uncleared apoptotic SMCs in *P. gingivalis-*infected plague were associated with impaired macrophage efferocytosis. Thus, by pre-processing macrophages with apoEVs derived from *P. gingivalis-*infected SMCs, the ability to efferocytose apoptotic cells declined clearly as compared with the counterparts, whereas downregulating miR-143/145 in apoEVs contributed to enhanced efferocytosis (Fig. S6a). Additionally, by co-culturing these apoEVs with macrophages, the ability of osteoclastic differentiation was obviously enhanced, but this tendency could be abolished by the miR-143/145 inhibitor (Fig. S6b). Next, to explore how miR-143/145 dysregulated macrophage efferocytosis, miR-143/145 inhibitor- or negative control-treated macrophages were subjected to RNA sequencing (RNA-seq). Gene set enrichment analysis revealed that the focal adhesion pathway was the most significantly downregulated pathway (Fig. S5b), which was associated with macrophage phagocytosis by regulating the actin cytoskeleton.^31,32^ We experimentally confirmed this result and found that focal adhesion kinase (FAK) phosphorylation was decreased after co-culturing with apoEVs, but increased after treatment with miR-143/145-inhibited apoEVs (Fig. S5c). Interestingly, heatmap analysis revealed that Siglec-G was markedly decreased in miR-143/145-inhibited macrophages (Fig. S7a). As an anti-phagocytic signal, Siglec-G (Siglec-10 in humans) was highly expressed in advanced atherosclerotic plagues (Fig. 4b) and positively associated with inflammasome-related genes (NLRP3), apoptosis-related genes (CASP2 and CASP4) and chemokine ligands (CCL2, CCL22, CCL24) in 109 plaque samples from atherosclerotic patients (Fig. S7b, c). We verified the role of Siglec-G in regulating macrophage efferocytosis using anti-Siglec-G antibodies and found that although miR-143/145 overexpression in macrophages reduced the clearance of apoptotic cells, Siglec-G blockade restored macrophage efferocytosis (Fig. 4c). Downregulated Siglec-G mRNA and protein in macrophages were further verified by the miR-143/145 inhibitor, but miR-143/145 mimics displayed the opposite result (Fig. S7d, e). These results suggest that miR-143/145 derived from apoptotic SMCs represses macrophage efferocytosis by increasing Siglec-G expression.

### Nuclear translocation of miR-143/145 activates Siglec-G transcription

Given that miR-143/145 are unlikely to positively control gene expression by the canonical RNA-induced silencing complex (RISC) in the cytoplasm, we hypothesized that miR-143/145 might exert non-canonical function in Siglec-G regulation, as previous studies have unveiled non-conventional regulatory functions of miRNA in gene transcription involving in cardiovascular homeostasis and pathology.^33,34^ Hence, we transfected RAW264.7 cells with fluorescently labeled miR-143/145 mimics and found that with the extension of incubation time, miR-143/145 was gradually translocated from the cytoplasm to the nucleus (Fig. 4d). Noticeably, miR-143/145 didn’t colocalize in the cytoplasm, but once translocated into nucleus, they aggregated at the same spatial location. Since Ago2 (Argonaute2), a key component of RISC, could preferentially interact with promoters to activate gene transcription, we investigated whether the Ago2-miR-143/145 complex might directly bind Siglec-G promoters to regulate its transcription. We first confirmed the positive regulation of Ago2 in Siglec-G expression via knocking down Ago2 in macrophages, revealing that the absence of Ago2 abolished the miR-143/145-enhanced Siglec-G expression (Fig. S8a). By comparing the miR-143/145 sequence in Siglec-G promoter regions, we obtained three putative binding sites by miR-143 (Sites a–c) and another three sites by miR-145 (Sites d–f) in Siglec-G promoters (Fig. S8b). Next, to ascertain the interaction of Ago2 in Siglec-G promoters, we designed 9 paired primers (upstream 2 kb sequences from the transcription start site) to cover the promoter region for ChIP-PCR assay and found a significant enrichment at Sites 1, 2, 3, 7, 9 (Fig. S8c). Then, these paired primers were further performed for Ago2-ChIP-PCR detection in miR-143- or miR-145-overexpressing macrophages. As shown in Fig. 4e, enrichment at Sites 1, 3, 7, and 9 was markedly increased in miR-143-overexpressing macrophages, but only Site 1 corresponded to miR-143 binding Site a (sequence TTCATCT). In miR-145-overexpressing macrophages, we observed a large enrichment at Site 2 that corresponded to miR-145 binding Sites d and e (sequence TGGGAAA). To test the functional significance of miR-143/145 in regulating Siglec-G transcription, Site a or Sites d and e were harbored in pGL3-Siglec-G 5′ a or pGL3-Siglec-G 5′ d and e plasmid, respectively. Consequently, miR-143 overexpression increased the fluorescence signal of the pGL3-Siglec-G 5′ a plasmid and miR-145 overexpression enhanced the signal of the pGL3-Siglec-G 5′ d and e plasmid. However, miR-143 or miR-145 inhibition diminished the fluorescence signal of the corresponding plasmid (Fig. 4f). To further illustrate the functional role of the Ago2-miR-143/145 complex in enhancing Siglec-G transcription, we constructed a pcDNA plasmid harboring a nuclear localization signal fused with Ago2 (Ago2nls). As macrophages were pretreated with si-Ago2 to ablated endogenous Ago2, exogenously nuclear Ago2 were indeed translocated into the nucleus (Fig. 4g) and promoted Siglec-G expression in the presence of miR-143 or miR-145 mimics (Fig. 4h). Meanwhile, we also designed a cytoplasmic Ago2 by adding a nuclear export signal after Ago2 (Ago2nes) and found that cytoplasmic Ago2 failed to facilitate Siglec-G expression (Fig. S8d, e), strongly supporting that nuclear Ago2-miR-143/145 complexes were responsible for activating Siglec-G transcription in macrophages. The Ago2-involved transcriptional activation has been reported to be associated with recruiting RNA polymerase II (POL II) to promoters.^35^ To verify these potential binding interactions in macrophages, we performed a co-immunoprecipitation (co-IP) assay in the presence of miR-143 or miR-145, revealing the direct physical colocalization between Ago2 and POL II (Fig. 4i). Together, these data suggest that nuclear miR-143/145 coupling with Ago2 activates Siglec-G transcription and then impairs macrophage efferocytosis.

### Influence of miR-143/145 deletion or overexpression on *P. gingivalis*-accelerated atherosclerosis

**Fig. 5:**
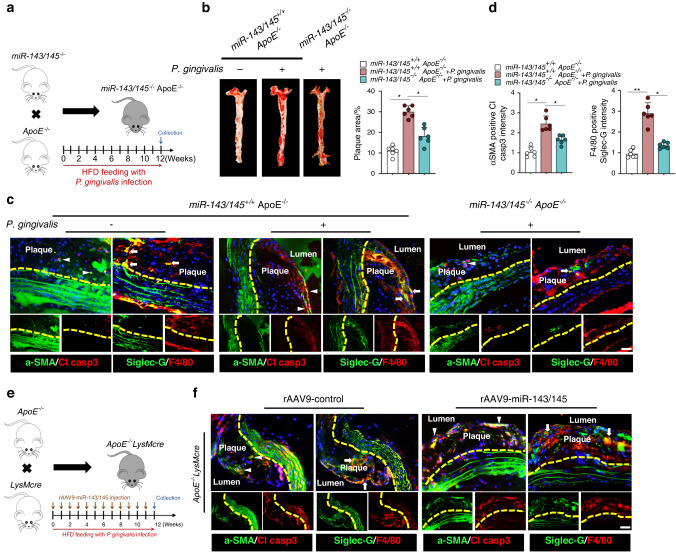
The in vivo role of miR-143/145 in *P. gingivalis*-infected animals. **a** Construction of *miR-143/145*^*−/−*^*ApoE*^*−/−*^ mice for 12 weeks HFD feeding, together with *P. gingivalis* infection or not. **b** Oil Red O staining showed the aorta en face in *miR-143/145*^*−/−*^*ApoE*^*−/−*^ or *miR-143/145*^*+/+*^*ApoE*^*−/−*^ mice, with quantitative data of plaque area at the right. *n* = 6. **c** Immunostaining staining of apoptotic SMCs (Cleaved caspase3^+^ α-SMA^+^ cells) and Siglec-G^+^ macrophages (Siglec-G^+^ F4/80^+^ cells) in serial plaque sections from *miR-143/145*^*−/−*^*ApoE*^*−/−*^ or *miR-143/145*^*+/+*^*ApoE*^*−/−*^ mice. Cl casp3 Cleaved caspase3. *n* = 6. Scale bars: 50 μm. **d** Quantitative analysis of apoptotic SMCs and Siglec-G^+^ macrophages in the aorta. **e** Construction of *ApoE*^*−/−*^*LysMcre*^*−/−*^ mice with *P. gingivalis* infection or not for 12 weeks HFD feeding and intravenously injected with rAAV9-control or rAAV9-miR-143/145 vector once a week. **f** Immunostaining staining of apoptotic SMCs and Siglec-G^+^ macrophages in serial plaque sections from *miR-143/145*^*−/−*^*ApoE*^*−/−*^ or *miR-143/145*^*+/+*^*ApoE*^*−/−*^ mice. The arrowhead indicates apoptotic SMCs. Arrow indicates Siglec-G^+^ macrophages. *n* = 6. Scale bars: 50 μm. Results are presented as the mean ± S.D. by one-way ANOVA followed by Tukey multiple comparisons tests. *P< 0.05; **P < 0.01

We next investigated the role of the miR-143/145-Siglec-G axis in developing *P. gingivalis*-accelerated atherosclerosis in vivo. In a *P. gingivalis*-infected *miR-143/145*^*−/−*^*ApoE*^*−/−*^ mouse model (Fig. 5a), alveolar bone loss was obviously accelerated, as evidenced by the bone parameters of BV/TV and BMD (Fig. S9a). Compared with that in *P. gingivalis*-infected *miR-143/145*^*+/+*^*ApoE*^*−/−*^ mice, miR-143/145 deficiency contributed to decreased osteoclasts on the bone surface (Fig. S9b) and reduced apoptotic cells in the periodontium (Fig. S9c). Since the macrophage efferocytosis could be assessed in vivo by detecting the spatial distribution of free apoptotic cells and macrophages,^17^ we observed increased co-localization of Cleaved caspase3 and F4/80 positive region in the periodontium in *miR-143/145*^*−/−*^*ApoE*^*−/−*^ mice, but not in *miR-143/145*^*+/+*^*ApoE*^*−/−*^ mice (Fig. S9c, indicated by the red box). For the vascular observation, there was a marked decrease of plaque area in both the aortic en face (Fig. 5b) and aortic roots (Fig. S9d) in *P. gingivalis*-infected *miR-143/145*^*−/−*^*ApoE*^*−/−*^ mice. By analyzing apoptotic SMCs (α-SMA^+^ Cleaved caspase3^+^ cells) and Siglec-G^+^ macrophages (Siglec-G^+^ F4/80^+^ cells) in serial plaque sections (Fig. 5c, d), we observed that the numbers of apoptotic SMCs and Siglec-G^+^ macrophages were higher in *P. gingivalis*-infected *ApoE*^*−/−*^ mice than in *ApoE*^*−/−*^ mice, whereas deleting miR-143/145 in *ApoE*^*−/−*^ mice potently reduced apoptotic SMCs and Siglec-G^+^ macrophages in the presence of *P. gingivalis*, indicating that macrophage efferocytosis was restored by downregulating miR-143/145. To further illustrate the effect of miR-143/145 on *P. gingivalis*-accelerated atherosclerosis in a macrophage-specific cell line, we constructed *ApoE*^*−/−*^*LysMcre* mice and generated a cre-dependent rAAV9-mediated miR-143/145 overexpression vector (rAAV9-miR-143/145) that was delivered into *ApoE*^*−/−*^*LysMcre* mice via tail vein (Fig. 5e). The efficiency of miR-143/145 overexpression in macrophages from rAAV9-miR-143/145-injected *ApoE*^*−/−*^*LysMcre* mice was determined by qRT-PCR, showing ~2-fold upregulation compared with the rAAV9-control group (Fig. S10a). By transducing rAAV9-miR-143/145 in *ApoE*^*−/−*^*LysMcre* mice, the interdental bone loss was further exacerbated between the first and second molars (Fig. S10b), which was accompanied by a more significant increase of osteoclasts and apoptotic cells as compared with rAAV9-control-treated *ApoE*^*−/−*^*LysMcre* mice (Fig. S10c, d). For aortic plaque detection, aggravated atherosclerotic lesion areas in the aorta tree and roots (Fig. S10e, f) were observed in rAAV9-miR-143/145-treated *ApoE*^*−/−*^*LysMcre* mice. Notably, transduction with rAAV9-miR-143/145 significantly elevated apoptotic SMCs and Siglec-G^+^ macrophages in serial plaque sections compared with their control counterparts (Figs. 5f and S10g). Collectively, the above results provide strong evidence that miR-143/145 deficiency protects against *P. gingivalis*-accelerated atherosclerosis, but macrophage-specific miR-143/145 overexpression aggravates atherosclerotic progression.

### Pretreated macrophage membranes to coat metronidazole and anti-Siglec-G antibody for precisely alleviating *P. gingivalis*-infected atherosclerosis

**Fig. 6:**
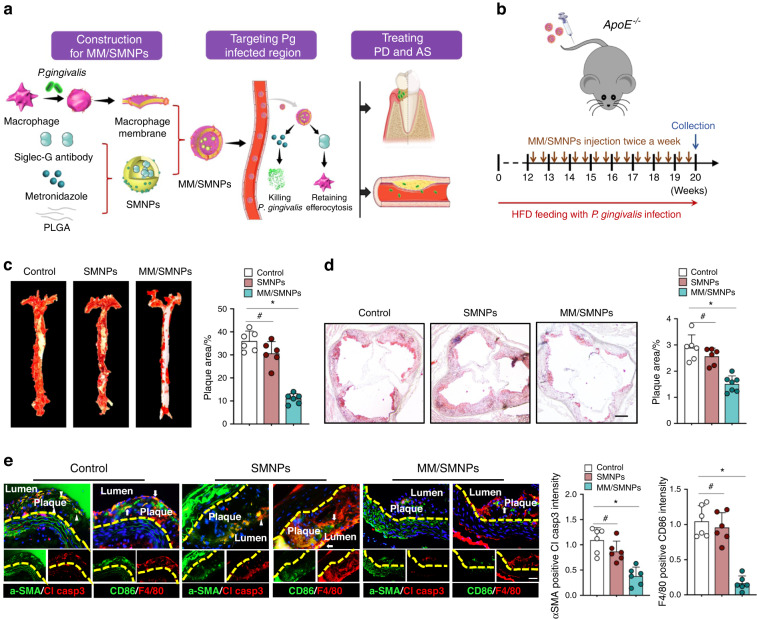
Fabrication of MM/SMNPs nanoparticles and its treatment for *P. gingivalis*-infected periodontitis and atherosclerosis. **a** Schematic illustration of SMNPs fabrication coated by *P. gingivalis-*pretreated macrophage membranes and delivery strategy in *P. gingivalis*-infected periodontitis (PD) and atherosclerosis (AS). **b** Schematic diagram of injection sites and frequency into *ApoE*^*−/−*^ mice. **c** Plaque area in an aortic tree and **d** in the aortic root was examined by Oil Red O staining. *n* = 6. Scale bars: 200 μm. **e** Immunostaining staining and quantitation of apoptotic SMCs and CD86^+^ macrophages in serial plaque sections from control, SMNPs, and MM/SMNPs injected *ApoE*^*−/−*^ mice. *n* = 6. Scale bars: 50 μm. Results are presented as the mean ± S.D. by one-way ANOVA followed by Tukey multiple comparisons tests. *P < 0.05; #*P* > 0.05

The above results demonstrated the adverse effect of *P. gingivalis* on inducing SMCs apoptosis and impaired macrophage efferocytosis in vivo and in vitro. Thus, therapeutic strategies based on antibacterial intervention and restoring immune clearance using metronidazole and anti-Siglec-G antibody might be a feasible approach to reduce the burden of *P. gingivalis*-accelerated atherosclerosis. Although anti-Siglec-G antibody and metronidazole could be encapsulated into PLGA-chitosan nanoparticles (SMNPs), the low drug concentration in the diseased area after injecting these nanoparticles into circulation^36^ urged us to develop a precise delivery system for targeting the *P. gingivalis*-infected region. Recently, macrophages have been considered as the first line against bacterial infection by expressing a series of receptors, in particular the toll-like receptors, to specifically bind microbial molecules.^37,38^ This means the possibility of exploiting macrophage membranes (MM) coatings as targeted delivery to bacterial infections (Fig. 6a). Inspired by this bacterial recognition ability, macrophages were pretreated with *P. gingivalis* to activate corresponding pathogen-related receptors, which were observed with enhanced expression of TLR2 and TLR4 on the MM (Fig. S11a). Then, these pre-treated MM were isolated for coating SMNPs nanoparticles to obtain MM/SMNPs with an average diameter of 350 nm (Fig. S11b). We found that the zeta potential of the MM/SMNPs was decreased as compared with that of antibody-loaded PLGA or metronidazole-loaded chitosan (Fig. S11c) and these MM/SMNPs nanoparticles remained stable in PBS over 7 days (Fig. S11d). To test the release kinetics of metronidazole and anti-Siglec-G antibody from MM/SMNPs, they were incubated in PBS for 14 days, revealing a 62.12% release of metronidazole (Fig. S11e) and 30.05% release of antibody (Fig. S11f). To verify whether MM/SMNPs could specifically bind to *P. gingivalis*-infected sites in vivo, MM/SMNPs were loaded with the fluorescent dye indocyanine green (ICG) and in turn, intravenously injected into *P. gingivalis*-infected *ApoE*^*−/−*^ mice. As monitored by fluorescence imaging after 24 h, fluorescence intensity was observed in the maxilla and aortic arch compared with that in the PBS-injected groups (Fig. S11g).

To explore the influence of MM/SMNPs on *P. gingivalis*-accelerated atherosclerosis, these nanoparticles were intravenously injected into *ApoE*^*−/−*^ mice twice a week after 12 weeks of HFD feeding (Fig. 6b). After 2 months treatment, the tissue morphology of the liver, spleen, and kidney was evaluated, and no obvious pathological changes were observed in SMNPs- or MM/SMNPs-injected mice (Fig. S12a). For micro-CT detection, even though there was a slight increase in alveolar bone in the SMNPs-treated groups, the difference was more evident in MM/SMNPs-injected mice (Fig. S12b), which was also accompanied by decreased osteoclast numbers and apoptotic cells in contrast with the control counterparts (Fig. S12c, d). Importantly, there was a considerable reduction in plaque area in the aortic tree (Fig. 6c) and diminished lipid deposition in the aortic root from MM/SMNPs-treated mice (Fig. 6d), suggesting the inhibitory effect of MM/SMNPs on delaying *P. gingivalis*-infected atherosclerosis. Furthermore, by serial plaque sections, we observed decreased apoptotic SMCs and downregulated proinflammatory macrophages (CD86^+^ F4/80^+^ cells) (Fig. 6e), probably owing to macrophage efferocytosis-involved polarization regulation.^39,40^ Taken together, our data indicate that the administration of metronidazole and anti-Siglec-G antibody coated on *P. gingivalis*-pretreated MM is an effective strategy for *P. gingivalis-*infected atherosclerosis.

## Discussion

**Fig. 7:**
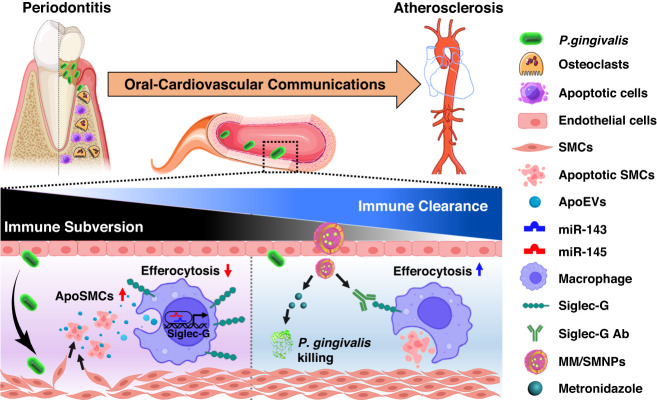
Model of *P. gingivalis*-induced immune subversion in atherosclerosis and the underlying therapeutical strategy. *P. gingivalis* that is orally implanted in the periodontal pockets can transfer into the blood circulation and ultimately colonize in inflammatory vascular sites. After crossing the endothelial cell layer, *P. gingivalis* facilitates SMCs apoptosis and extracellular release of apoEVs-miR-143/145. These apoEVs containing large amounts of miR-143/145 are captured by macrophages and trigger blocked efferocytosis by transcriptional enhancing Siglec-G expression to activate the anti-phagocytic signal, leading to accumulated apoptotic SMCs. To therapeutically kill bacteria and remove apoptotic SMCs, MM/SMNPs nanoparticles are developed and demonstrated to efficiently target *P. gingivalis-*infected periodontium and atherosclerosis, which subsequently exerts bactericidal effect and restores macrophage efferocytosis

*P. gingivalis* can execute its immune-subversive ability by inducing cellular apoptosis to promote its fitness in both periodontal tissue and atherosclerotic lesions.^41–44^ However, whether the accumulation or failed clearance of apoptotic cells in *P. gingivalis*-infected tissues is associated with disrupted macrophage efferocytosis remains elusive. In this study, from the view of oral-cardiovascular communication, we provided the molecular explanation for *P. gingivalis*-induced SMCs apoptosis and the inability of macrophage efferocytosis, and further developed functionalized biomimetic nanoparticles to precisely treat *P. gingivalis-*accelerated atherosclerosis. Specifically, SMCs exposed to *P. gingivalis* were sensitive to apoptosis and released a variety of apoEVs-miR-143/145 into the extracellular environment by activating TLR2 pathway. As specifically captured by macrophages, miR-143/145 is translocated into the nucleus to promote Siglec-G transcription, thus robustly suppressing macrophage efferocytosis. To resolve the *P. gingivalis-*accelerated atherosclerosis, MM/SMNPs were fabricated by coating *P. gingivalis*-pretreated MM onto metronidazole and anti-Siglec-G antibody-loaded nanoparticles to precisely kill *P. gingivalis* and restore macrophage efferocytosis ability (Fig. 7).

Interestingly, SMCs displayed greater sensitivity to *P. gingivalis*-induced apoptosis than ECs. Indeed, *P. gingivalis* not only invades but also activates aortic ECs by enhancing adhesion molecules and chemokines, thus accelerating the transmigration of *P. gingivalis*-hijacked leukocytes to the vascular wall and harboring *P. gingivalis* into deeper tissues, where they might directly induce SMCs apoptosis.^45^ Emerging evidence supports the notion that the event of increased apoptotic SMCs is a strongly detrimental factor in promoting plaque destabilization and atherosclerosis development.^46,47^ Therefore, our results suggested that *P. gingivalis*-induced SMCs apoptosis is probably the main reason for accelerating the plaque formation in *P. gingivalis*-infected condition. Although it has been well established that miR-143/145 is enriched in SMCs and beneficial to prevent SMCs de-differentiation or phenotype switching,^48^ our data showed that SMCs apoptosis occurs earlier than the significant changes of miR-143/145, revealing that the increased miR-143/145 expression has little biological significance for SMCs functions. In addition, given that cellular apoptosis is usually accompanied by massive releasing of apoEVs that are contained with many miRNAs cargos,^49,50^ we characterized the SMCs-derived apoEVs and found that apoEVs loaded with miR-143/145 could be assuredly captured by macrophages. Apart from the modulation of macrophage efferocytosis, we also found the decreased expression of matrix metalloproteinase 9 (MMP-9), an inflammatory mediator of atherosclerosis,^51^ in miR-143/145 inhibitory macrophages (Fig. S7a), further suggesting the beneficial effect on reduction of atherosclerosis by downregulating miR-143/145 in macrophages.

Recently, growing evidence has delineated the non-canonical paradigm of miRNAs in intracellular localization and transcriptional regulation that are relevant to cardiovascular diseases.^34,52^ In contrast with canonical localizations in the cytoplasm, several miRNAs, together with AGO proteins, are detectable in the nucleus.^53^ The availability of nuclear localization is probably associated with the specific sequence or motifs, such as the core GU-rich sequence for miR-126-5p, the ASUS motif (where S = C or G), and the 3′-end motif of AGUGUU for miR-141-5p.^34^ These nuclear miRNAs are capable of directly promoting gene transcription; for example, in the nucleus, miR-320 enhances CD36 transcription via Ago2-activated promoters in cardiomyocytes^33^ or recruits Ago1, EZH2, and tri-methyl histone H3 lysine 27 (H3K27me3) on the POLR3D gene promoter to epigenetically control gene transcription in mammalian cells.^54^ In our study, we observed the phenomenon of nuclear miR-143/145 aggregation in both SMCs and macrophages, which might result from their high GU-rich sequence of miR-143/145. Additionally, we demonstrated that nuclear miR-143/145 could enhance Siglec-G transcription by directing Ago2 to gene promoters. Consistent with this finding, our previous work has also characterized miR-143/145 as nuclear miRNA to activate SOX2 transcription by Ago2-modified H3K9me3 in bone mesenchymal stem cells (BMSCs).^55^ Here, we further demonstrated the transcriptional regulatory mechanism of nuclear miR-143/145 in activating the anti-phagocytic signal of macrophages and linked this manipulation to *P. gingivalis*-promoted atherosclerosis.

In periodontitis, oral pathogen invasion mediates miRNAs expression to subvert numerous innate immune responses and trigger inflammatory bone resorption, leading to osteoclastic overactivation.^56^ Despite knowing that *P. gingivalis*-induced miRNAs alternation is responsible for activated osteoclast differentiation,^56^ most observations mainly focused on the influence of the miRNAs produced by macrophages/osteoclasts themselves with *P. gingivalis* exposure but lacked of the investigation of extracellular miRNAs in osteoclast differentiation. Here, in our study, we found that apoEVs-miR-143/145 from *P. gingivalis-*infected SMCs could be captured by macrophages and promote osteoclast differentiation. Furthermore, we found that deleting miR-143/145 in *ApoE*^*−/−*^ mice reduced osteoclast numbers and bone resorption, while overexpression of miR-143/145 in macrophages exacerbated bone loss, confirming the inhibitory role for osteoclast differentiation by downregulating miR-143/145 in *P. gingivalis*-promoted atherosclerosis. We previously showed that miR-143/145 were highly expressed in estrogen-deficient women, and *miR-143/145*^*−/−*^ mice prevented ovariectomized-induced osteoclast overactivation and bone loss.^55^ Since estrogen deficiency could also increase atherosclerosis risk,^57^ this evidence supports a potential relationship between miR-143/145 and atherosclerosis in menopausal women, but further studies are needed to evaluate the underlying mechanism.

Clinical interventional studies and meta-analyses suggest that periodontal treatment, a debridement that mechanically and potently removes supra- and subgingival bacteria, reduces systemic inflammation and improves the atherosclerotic profile.^58–60^ Although it is possible to reduce atherogenic risk by downregulating *P. gingivalis*, periodontal treatment could not relieve the existing atherosclerotic plaques.^58,61^ Currently, clinical treatment of atherosclerosis is aimed to inhibit lipid and platelet aggregation, but those drugs could not fully resolve this problem, which might be attributed to multiple pathogenetic factors and low drug concentration local.^62,63^ A previous study developed MM loaded with rapamycin for targeted AS therapy by exploiting the natural features of macrophages of “homing” into inflammatory diseases (e.g., atherosclerotic lesions).^64^ In our study, to improve targeting to *P. gingivalis*-infected plaques, macrophages were pretreated with *P. gingivalis* infection and then subjected to membrane isolation. Considering the contradictory reports of miR-143/145 roles in atherosclerotic progression, we employed an antibody blockade strategy to increase macrophage efferocytosis. With coupled administration of anti-Siglec-G antibody and metronidazole that were loaded into *P. gingivalis*-pretreated MM, we observed that the nanoparticles could precisely target periodontal tissue and atherosclerotic lesions (Fig. S11g). Although a relatively low intensity was observed in the periodontium or aortas compared with the metabolic organs of the liver and kidney, no fluorescent signal could be detected in the other non-*P. gingivalis*-infected regions such as the cranium, femur, or spleen. In addition, with the constant injection of these drugs into *ApoE*^*−/−*^ mice for 2 months, the nanoparticle accumulation would be definitely strengthened in periodontium and aortas. Together, based on the concept of anti-bacteria and immune clearance, our data might provide a promising drug delivery system for treating both periodontitis and *P. gingivalis*-accelerated atherosclerosis.

Although our work uncovered several important findings, some limitations need to be also pointed out. Firstly, using cell type-specific transgenic mice could be more rigorous to support our conclusion. Secondly, considering the different phenotypes and functions of SMCs in vascular media and intima, the role of *P.gingivalis* on SMCs phenotype switch is worthy of in-depth study. Lastly, in the context of *P. gingivalis*-induced periodontitis of WT mice, miR-143/145 levels exhibited a significant elevation in periodontal tissue and blood circulation, but not in aortic root tissue (Fig. S1a). However, we cannot rule out the possibility that circulatory miR-143/145 in *P. gingivalis*-infected *ApoE*^*−/−*^ mice may further deteriorate atherosclerotic plaque progression. Therefore, following studies targeting *P. gingivalis*-induced circulatory indicator changes (e.g., protein, metabolites, or miRNAs) would contribute to better defining the influence of *P. gingivalis* in promoting atherosclerotic progression.

The ability of *P. gingivalis* to subvert the host immune response for its survival not only locally disrupts the periodontium but is also systemically linked to cardiovascular diseases. Our study provides new insights into the molecular mechanism of *P. gingivalis*-accelerated atherosclerosis and develops a promising therapeutic strategy to achieve precise and effective treatment.

## Materials and methods

### Human samples

Human gingival tissues were harvested from healthy volunteers or patients who suffered from chronic periodontitis when they underwent dental treatment at the affiliated hospital of stomatology of Nanjing medical university (Approval No. PJ2020-126-001). The inclusion criteria for chronic periodontitis were in the presence of bleeding on probing, diseased sites with clinical attachment loss ≥4 mm associated with probing pocket depth ≥6 mm, and radiographic evidence of bone loss. All subjects included in the study have written informed consent prior to sample collection through the approval of the Ethics Committee of the School of Stomatology of Nanjing Medical University.

### Mice

All animal experiments were performed with the consent of the Ethics Committee of the School of Stomatology of Nanjing Medical University. All procedures were conducted in accordance with the guidelines of the Animal Care Committee of Nanjing Medical University (Approval No. IACUC-2006014). *miR-143/145*^*−/−*^*ApoE*^*−/−*^ mice were generated by crossbreeding *miR-143/145*^*−/−*^ (Model Animal Research Center of Nanjing University, T001458) and *ApoE*^*−/−*^ mice (Model Animal Research Center of Nanjing University, T000090). *TLR2*^*−/−*^*ApoE*^*−/−*^ mice were generated by crossbreeding *TLR2*^*−/−*^ (Model Animal Research Center of Nanjing University) and *ApoE*^*−/−*^ mice. *ApoE*^*−/−*^ mice were mated with *LysMcre* mice (Model Animal Research Center of Nanjing University, N000056) to generate *ApoE*^*+/−*^*LysMcre* heterozygous mice. Then we obtained *ApoE*^*−/−*^*LysMcre* mice by crossing *ApoE*^*+/−*^*LysMcre* mice and *ApoE*^*−/−*^ mice. The mice genotypes were verified by PCR using genomic DNA from the tail. Mice were housed in ventilated cages and had access to food and water ad libitum.

### Animal experiments

To induce atherosclerosis, all male mice at 6-week-old in this study were randomly grouped and fed with a high-fat diet (HFD) containing 1.25% cholesterol (Research Diets D12108C) for 12 weeks. Together with HFD feeding, *P. gingivalis* were orally inoculated on the bilaterally buccal mucosa of maxillary molar in each littermate (10^8^ CFU in 0.1 mL 2% carboxymethylcellulose with phosphate-buffered saline) five times a week for 12 weeks. Cre-dependent recombinant AAVs (serotype rAAV9, vector: pAAV-CMV bGlobin-FLEX-eGFP-WPRE-hGH polyA, Cat# AAV9-62648-1 and AAV9-62647-1) were purchased from GeneChem to make miR-143/145 overexpression in LysM-lineage cells. *ApoE*^*−/−*^*LysM*cre mice were injected with rAAV9-miR-143/145 (6.75 × 10^12^  virus genomes per mL) or control rAAV9 (5.2 × 10^12^ virus genomes per mL) once a week for 12 weeks at a dose of 10 μL) by tail vein injection. The overexpressing efficiency of miR-143/145 was evaluated by inducing primary bone marrow cells into macrophages. Therapeutically, after 12 weeks of HFD feeding together with *P. gingivalis* infection, the MNPs and MM/SMNPs were intravenously injected into *ApoE*^*−/−*^ mice at an ST-equivalent dosage of 1.6 mg/kg twice a week for 8 weeks. Mice were sacrificed at the indicated time points and tissues of the maxilla, heart, and thoracic abdominal aorta were collected and fixed in 4% paraformaldehyde (PFA) for further analysis (*n* = 6 per group).

### Bacteria culture and detection

*Porphyromonas gingivalis* (ATCC 33277), *Aggregatibacter actinomycetemcomitans* (ATCC 29523), *Prevotella intermedia* (ATCC 25611) strains were inoculated onto blood agar plates and anaerobically cultured at 37 °C for 5–7 days. Then *P. gingivalis* strains were grown anaerobically in brain heart infusion broth supplemented with hemin (5 μg·mL^−1^) and vitamin K1 (5 μg·mL^−1^) for over 24 h at 37 °C. The total DNA in atherosclerotic plaque from *ApoE*^*−/−*^ mice was detected using a DNA extraction kit (Tiangen, China). The 16S ribosomal-RNA gene of *P. gingivalis* was amplified by specific bacterial primers, whose sequences were listed in Supplementary Table 3.

### Cell culture and infection with *P. gingivalis*

The mouse aortic EC line, mouse aortic SMCs line, mouse macrophage cell line (RAW 264.7), and mouse fibroblast cells (L929) were inoculated in DMEM medium containing 10% fetal bovine serum (FBS), 100 U·mL^−1^ penicillin and 100 mg·mL^−1^ streptomycin. The cells were cultured at 37 °C with 5% CO_2_, and the medium was changed every 2 days. Mouse bone marrow mesenchymal stem cells (BMSCs) were flushed from the bone marrow cavities in femurs and tibias using DMEM containing 2% FBS and 100 U·mL^−1^ penicillin and 100 mg·mL^−1^ streptomycin. Bone marrow cells were cultured in a complete medium with no interference for 3 days and then nonadherent cells were removed by replenishing the culture medium. After three passages, the purified BMSCs were obtained for the following assay. We obtained primary macrophages by exploiting bone marrow cells onto 100-mm Petri dishes for 4 h incubation and collected nonadherent cells in complete DMEM with M-CSF (10 ng·mL^−1^) for 3 days. For the co-culture assay, cells were seeded in 6-well plates overnight and then co-incubated with *P. gingivalis* for 24 h at 37 °C. After that, infected cells were washed three times with PBS to remove external, non-adherent *P. gingivalis* and harvested for follow-up experiments.

### Microcomputed tomography (micro-CT) analysis

The micro-architectural properties of the maxillae were analyzed using the micro-CT system (Skyscan 1176, Kontich, Belgium). The maxillae were scanned at an energy of 50 kV and 456 μA and a standard resolution of 18 μm. The maxillae samples were reconstructed using the SkyScan NRecon v.1.6 programs and analyzed using SkyScan CTAn v.1.13.8.1 software (SkyScan). The region of interest (ROI) was focused on the interdental bone between the first and second maxilla molars. To evaluate the bone structure, the following parameters were calculated: bone volume and BMD.

### Oil red O staining and hematoxylin-eosin staining

The mouse heart and aorta were perfused, dissected, and cleaned from surrounding fat tissue. The aorta was fixed with 4% PFA, opened longitudinally, pinned flat onto the plate, and stained with Oil Red O (Beyotime, Shanghai, China). The percentage of the area of red stained lipids with Oil Red O to the total area is calculated to analyze lipid deposition. The mouse hearts and the vascular strips were immersed in 20% sucrose solution for 24 h, embedded in OCT, and then cut into tissue sections (serial sections, 6 μm). The serial sections were cut from the onset of the aortic valves and then stained with Oil Red O staining. The quantification of the atherosclerotic lesion area in the full-length aorta and aortic root lesion area stained with Oil Red O was quantitated using the Image J software. The mouse liver, spleen, and kidney were collected and fixed with 4% PFA. After being immersed in 20% sucrose solution for 24 h, they were embedded in OCT and cut into 6 μm tissue sections. Sections were stained with hematoxylin-eosin staining.

### Immunofluorescence and trap staining

For immunofluorescence, the maxillae were fixed with 4% PFA and decalcified in 10% EDTA for 6 weeks. The decalcified murine maxillae and the atherosclerotic plaque tissue were immersed in 20% sucrose solution for another 24 h and embedded in OCT. Then 4-μm-thick sections were cut for staining using a cryostat microtome (Leica, Wetzlar, Germany). The tissue slices were washed with PBS and permeabilized with 1% Triton X-100 for 15 min. After blocking with goat serum, the sections were incubated with the primary antibodies overnight at 4 °C. Detailed information about the primary antibodies is listed in Supplementary Table 4. The sections were washed with PBS for 3 times and stained with FITC or Cy3-labeled secondary IgG at 37 °C for 1 h and labeled with DAPI for 3 min at room temperature. The sections were captured using fluorescence microscopy (Leica Microsystems, Mannheim, Germany).

For histological staining, the maxillae were dehydrated, embedded in paraffin wax, and sectioned into 4-μm-thick slices. Osteoclasts on the alveolar bone surface between the first and second maxilla molars were visualized by tartrate-resistant acid phosphatase (TRAP) staining kit (Sigma Aldrich, St Louis, MO, USA) according to the manufacturer’s instructions and quantified by calculating osteoclast number per bone surface (Oc. N/BS).

### Fluorescence in situ hybridization (FISH) and TUNEL assay in vivo and in vitro

FISH probes were directly labeled with Fluorescent In Situ Hybridization Kit (Genepharma, Shanghai, China) according to the manufacturer’s instructions, and the probes were designed and synthesized by Genepharma (Shanghai, China). The section was added with Proteinase K and incubated at 37 °C for 20 min. Then, each tissue section was washed by 2× SSC wash buffer at room temperature for 3 times and rehydrated through an alcohol gradient of 2 min each (70% alcohol, 80% alcohol,90% alcohol, 100% alcohol). Next, 100 μL of pre-warmed denaturation solution was applied to the sample and incubated for 8 min at 78 °C and then the section was rehydrated through an alcohol gradient again. After that, the tissue was incubated with FISH probes (miR-143, 5ʹ-GAGCTACAGTGCTTCATCTCA-3ʹ; miR-145, 5ʹ-AGGGATTCCTGGGAAAACTGGAC-3ʹ) at 37 °C for 12–16 h. The section was washed with a pre-warmed 2× SSC wash buffer for 15 min. After washing with PBS for 10 min, the section was labeled by DAPI (2 μg·mL^−1^) for 5 min at room temperature and images were captured under a fluorescence microscope (Leica Microsystems, Mannheim, Germany). For the in vitro detection, the prepared cells seeded on coverslips were fixed with 4% paraformaldehyde for 30 min. Then the coverslips were permeabilized with 0.1% Triton X-100 for 15 min and washed with 2× SSC for 30 min at 37 °C. The cells were incubated with miR-143/145 FISH probes at 37 °C overnight. Subsequently, cells were stained with Hoechst 33342 for 3 min and then detected by fluorescence microscope.

For the detection of apoptotic cells in tissues, the TUNEL assay was performed following the manufacturer’s instructions using TUNEL BrightGreen Apoptosis Detection Kit (A112-02, Vazyme, Nanjing, China). Briefly, the frozen tissue sections were washed with PBS and permeabilized with 0.2% Triton X‐100 for 10 min. After blocking in 5% BSA for 1 h at room temperature, sections were allowed to equilibrate with 1× Equilibration Buffer at room temperature for 20 min. Then each section was incubated with terminal deoxynucleotidyl transferase (TdT) enzyme solution (34 μL ddH_2_O + 10 µL 5× Equilibration Buffer +5 µL BrightGreen Labeling Mix + 1 μL Recombinant TdT Enzyme) at 37 °C for 60 min. The sections were washed with PBS for 3 times and stained with DAPI (2 μg·mL^−1^) for 5 min. The cells showing green fluorescence were considered apoptotic cells and the images were captured using a fluorescence microscope. For the in vitro apoptotic detection, when infected with *P. gingivalis*, cells on the slide were washed with PBS for 3 times and fixed with 4% paraformaldehyde for 30 min. Then the slide was treated with 20 μg·mL^−1^ of Proteinase K for 5 min, incubated with Equilibration Buffer for 10–20 min, and TdT enzyme solution for 60 min. The cells were stained with Hoechst 33342 and observed by fluorescence microscope.

### Western blot and co-immunoprecipitation

The cells were lysed in RIPA buffer (Beyotime, Shanghai, China) containing 10 mM protease inhibitor (PMSF; Beyotime) and 1% protease inhibitor cocktail for 30 min on ice. Protein lysate was loaded onto SDS-PAGE gels and transferred to the PVDF membrane (Millipore, Billerica, MA, USA). The membranes were blocked with 5% defatted milk for 2–3 h at room temperature and incubated with primary antibodies at 4 °C overnight. Detailed information about the primary antibodies is listed in Supplementary Table 4. The membranes were washed with TBST for 3 times and incubated with the corresponding HRP-conjugated secondary antibodies (1:8 000) for 1 h. Finally, the images of protein bands were visualized by the Tanon detection system using the enhanced chemiluminescence reagent (Thermo Scientific).

For co-IP assay, the cells were lysed by IP lysate buffer (P0013, Beyotime Biotechnology, Haimen, China), containing 1% Halt Protease & Phosphatase Inhibitor Cocktail (78445, Thermo, USA) on ice for 30 min and centrifuged to get the cell lysate. The supernatant was collected and transferred into new Eppendorf tubes for immunoprecipitation. The supernatant added with 50 μL of protein A/G agarose beads (Roche, Mannheim, Germany) was incubated with the antibody under constant rotation at 4 °C overnight. The mixture was washed 3 times with 1× washing buffer. The beads were resuspended in 1× SDS loading buffer, boiled for 10 min, and performed to Western blot assay. The antibodies used in this study are listed in Supplementary Table 4.

### RT-qPCR and ChIP-qPCR assays

The nuclear and cytoplasmic fractions were separated using a nuclear and cytoplasmic extraction kit (BB-36021, BestBio, Shanghai, China) according to the manufacturer’s instructions. Total RNA from the nuclear and cytoplasmic fractions or whole-cell lysates was extracted using an RNA extraction kit (RP1202, BioTeke, Beijing, China). Reverse transcription was performed with the PrimeScript RT Reagent Kit (Takara Bio, Kusatsu, Japan) according to the manufacturer’s recommendations. miRNAs were extracted from purified apoEVs using the miRNeasy Serum/Plasma kit (QIAGEN, Valencia, CA, USA) according to the manufacturer’s instructions. The mature miRNAs were detected by the All-in-One miRNA RT-qPCR Detection Kit (GeneCopoeia, Rockville, MD, USA). The levels of each miRNA were normalized to the U6 levels, and the miRNAs levels from apoEVs were normalized to the levels of spiked-in ce-miR-39 by using miRNeasy Serum/Plasma Spike-In Control kit (QIAGEN, Valencia, CA, USA).

ChIP assay was conducted following the manufacturer’s instructions using the SimpleChIP Plus Sonication Chromatin IP Kit (#56383; Cell Signaling Technology, Danvers, MA). Briefly, RAW 264.7 macrophages were collected, crosslinked, and lysed. The chromatin pellets were sonicated to yield DNA fragments in ChIP dilution buffer. The supernatant was diluted with ChIP dilution buffer and then incubated with the primary antibody overnight at 4 °C under constant rotation. The antibodies used were shown in Supplementary Table 4. Then, the protein G magnetic beads were added to each sample and incubated for 2 h at 4 °C with rotation. Subsequently, the chromatin was eluted in ChIP Elution Buffer and the cross-link was reversed at 65 °C for 2 h. The DNA was isolated and purified for qPCR enrichment. The primer sequences used were listed in Supplementary Table 3.

### Phagocytosis assay

To examine the efferocytosing ability of macrophages in different conditions, SMCs pre-stained with CFSE (Cat# 65-0850-84, Invitrogen) were exposed to transient ultraviolet light for 30 min to induce apoptosis and co-cultured with DiI (Cat# 40726ES10, Yeasen) labeled macrophages. in equal numbers (1:1) for 3 h. Then, after removing the free cells, the remaining macrophages were fixed with 4% paraformaldehyde labeled with Hoechst 33342 for 3 min. When observed by fluorescence microscope, the macrophages showing simultaneously green and red fluorescence were considered as efferocytosing macrophages.

### Transfection and Luciferase reporter assay

miRNA mimics and inhibitors were synthesized and purchased by GenePharma Co. Ltd. (Shanghai, China). Cells were seeded in 24-well plates overnight and transfected with miRNA control, miR-143/145 mimics or miR-143/145 inhibitors, Renilla vector (pRL-TK; Promega, Madison, WI, USA), pGL3-basic luciferase reporter vector containing the predicted region of Siglec-G promoter by using Lipofectamine 2000 (Invitrogen). The interference sequences and reporter vector are listed in Supplementary Table 3. Finally, after 48 h incubation, firefly and renilla luciferase activity were measured using the Dual-Luciferase Reporter Assay (Promega, Madison, WI, USA). The firefly luciferase activity of each sample was normalized to renilla luciferase activity.

### Isolation and labeling of apoEVs or EC-Exos

To collect apoEVs from the apoptotic SMCs, the SMCs were infected with *P. gingivalis* for 24 h. After removing *P. gingivalis*, the remaining cells were washed 3 times using PBS and then cultured in serum-free DMEM for 48 h. The cell supernatant was harvested and centrifuged at 300×*g* for 10 min to remove cell lysate and the resultant supernatant was centrifuged at 2 000×*g* for 15 min to remove cell debris. Finally, the supernatant was ultracentrifuged at 100 000×*g* for 70 min twice to isolate the apoEVs. Vesicle pellets were resuspended with 100 µL PBS, aliquoted, and stored at −80 °C for subsequent experiments.

To verify the uptake of apoEVs by macrophages, apoptotic SMCs were stained with DiO cell-labeling solution (Cat# 40725ES10, Yeasen) according to the manufacturer’s instructions. Then, the obtained apoEVs were labeled DiO and co-cultured with macrophages for 3 h. Finally, the cells were fixed with 4% paraformaldehyde, labeled with Hoechst 33342, and observed by fluorescence microscope.

### Preparation of the MM

The MM of RAW264.7 was obtained using the Membrane Protein Extraction Kit (Beyotime Biotechnology, Shanghai, China) according to a previous study.^64^ Briefly, cells were cultured in 100 mm plates at a density of 1 × 10^6^ cells per plate overnight and then infected with 5 × 10^7^ CFU *P. gingivalis* for 3 h. The collected cells were resuspended in membrane protein extraction buffer solutions and incubated for 15 min at 4 °C. The sample was homogenized approximately 30 times using a glass homogenizer and centrifuged at 700×*g* for 10 min. Subsequently, the suspension was centrifuged at 4 °C at 14 000×*g* for 30 min to precipitate the cell membranes. The final cell membranes were sonicated for 15 min and extruded a minimum of 10 times through 200 nm pore-size polycarbonate filters (Avestin Inc., Ottawa, Canada). The pretreated MM was stored in water at 4 °C for the following experiments.

### Preparation of Sigle-G antibody and metronidazole-loaded nanoparticles (SMNPs)

SMNPs were prepared using the nano-precipitation method according to previous studies with slight modifications.^64,65^ Briefly, polyethylene glycol (PEG, 120 mg per 500 µL) solution was mixed with Siglec-G antibody (20 µg) for 30 min at room temperature to form the PEG-Siglec-G solution. Then, 50 mg PLGA, 15 mg Chitosan, 50 mg metronidazole and PEG-Siglec-G solution were dissolved in 5 mL acetic acid to obtain the PLGA-Siglec-G-chitosan-metronidazole nanoparticles (SMNPs) solution. Next, the mixture was emulsified using a sonicator to remove acetic acid. The PLGA-SMNPs were harvested by centrifugation and re-suspended in PBS.

### Preparation of MM-coated SMNPs (MM/SMNPs)

MM/SMNPs were fabricated by coating SMNPs with pretreated MMs through the extrusion method. Briefly, pretreated MM vesicles and SMNPs were mixed at a ratio of 1:1 and sonicated for 3 min. Then, the mixture was extruded 7–10 times through a mini-extruder with a 200 nm polycarbonate porous membrane (Avestin, LF-1, Canada) to collect the MM/SMNPs. The prepared MM/SMNPs were stored in PBS at 4 °C for further study. For in vivo tracing experiments, pretreated MMs and SMNPs nanoparticles were dissolved in 1 mL deionized water and fluorescently loaded with 1 mg/mL aqueous solution of indocyanine green (ICG; I2633, Sigma-Aldrich). The solution was sonicated by a sonicator bath and then extruded using the extruder to obtain ICG-loaded MM/SMNPs.

### Characterization of the MM/SMNPs

To observe the morphology, MM/SMNPs solution was deposited on a glow-discharged carbon-coated grid and immediately stained for 1 min with 1% phosphotungstic acid (pH 7.0), which were subsequently subjected for scanning electron microscopy (SEM, JEOL, JSM-6700F, Japan) detection. The zeta potential of Chitosan-metronidazole (CS-MTZ), PLGA-Siglec-G, SMNPs, and MM/SMNPs was determined using the Malvern zeta sizer (Nano ZS90, Malvern, UK). The mean particle size was measured for 6 days by Malvern zeta sizer to determine the MM/SMNPs stability. To investigate the release of metronidazole in MM/SMNPs, the MM/SMNPs were kept in PBS for 14 days at 37 °C and detected by UV/Vis spectrophotometer (UV-2450, Shimadzu, Japan) at different time points. For measuring the release of the Siglec-G antibody, the protein concentration of the supernatant was determined using the BSA protein assay kit (Thermo Scientific), and the antibody released rate was determined by dividing the amount of released protein by total protein.

### Statistical analysis

All data in the text are expressed as means ± SD. All these experiments above were repeated independently at least three times. Statistical significance was determined using Student’s *t*-test for two groups comparison. One-way analysis of variance (ANOVA) was performed for multiple comparisons. *P* < 0.05 (*), *P* < 0.01 (**) and *P* < 0.001 (***) was considered statistically significant. *P* > 0.05 was considered not significant.

## Data and materials availability

All data are available in the main text or the Supplementary materials.

## Supplementary information

Supplementary Material

Supplementary Table 1-Differential_analysis_results

Supplementary Table 2-GSEA

Supplementary Table 3-primer, interference, and plasmid sequence

Supplementary Table 4-antibody list

## Data Availability

The bioinformatics analysis in this paper was from Gene Expression Omnibus (GEO) database. TLR2 and Siglec-10 expression was analyzed in human early and advanced atherosclerotic plaque (GSE28829). Correlation analyses were obtained in 109 atherosclerotic patients (GSE23304).
