# Supplementary material for: Oral pathogen aggravates atherosclerosis by inducing smooth muscle cell apoptosis and repressing macrophage efferocytosis
Source: Int J Oral Sci. 2023 Jun 28;15:26. doi: 10.1038/s41368-023-00232-5 (PMC10307898; doi:10.1038/s41368-023-00232-5)
Supplement: Supplementary file 1 — Supplementary Material [file 41368_2023_232_MOESM1_ESM.pdf]

Supplementary Figures for

Oral pathogen aggravates atherosclerosis by inducing smooth muscle cells apoptosis and repressing macrophages efferocytosis

Authors: Hanyu Xie[1,2,4#], Ziyue Qin[2,3,4#], Ziji Ling[1,2,4], Xiao Ge[1,2,4], Hang Zhang[1,2,4], Shuyu Guo[2,4,5], Laikui Liu[2,4], Kai Zheng[2,4], Hongbing Jiang[1,2,4*], Rongyao Xu[1,2,4*]

Affiliations:

[1]Department of Oral and Maxillofacial Surgery, Affiliated Hospital of Stomatology, Nanjing Medical University, Nanjing 210029, China

[2]Jiangsu Key Laboratory of Oral Diseases, Nanjing Medical University, Nanjing 210029, China

[3]Department of Periodontology, Affiliated Hospital of Stomatology, Nanjing Medical University, Nanjing 210029, China

[4]Jiangsu Province Engineering Research Center of Stomatological Translational Medicine, Nanjing Medical University, Nanjing 210029, China

[5]Department of Orthodontics, Affiliated Hospital of Stomatology, Nanjing Medical University, Nanjing 210029, China.

[#]Hanyu Xie and Ziyue Qin contributed equally to this work.

[*]Corresponding authors: Rongyao Xu, rongyaoxu@njmu.edu.cn; Hongbing Jiang, jhb@njmu.edu.cn
Jiangsu Key Laboratory of Oral Diseases, Nanjing Medical University, 140 Hanzhong Road, Nanjing, Jiangsu Province 210029, China.

Tel: +86-25-85031914

Fax: +86-25-85031910

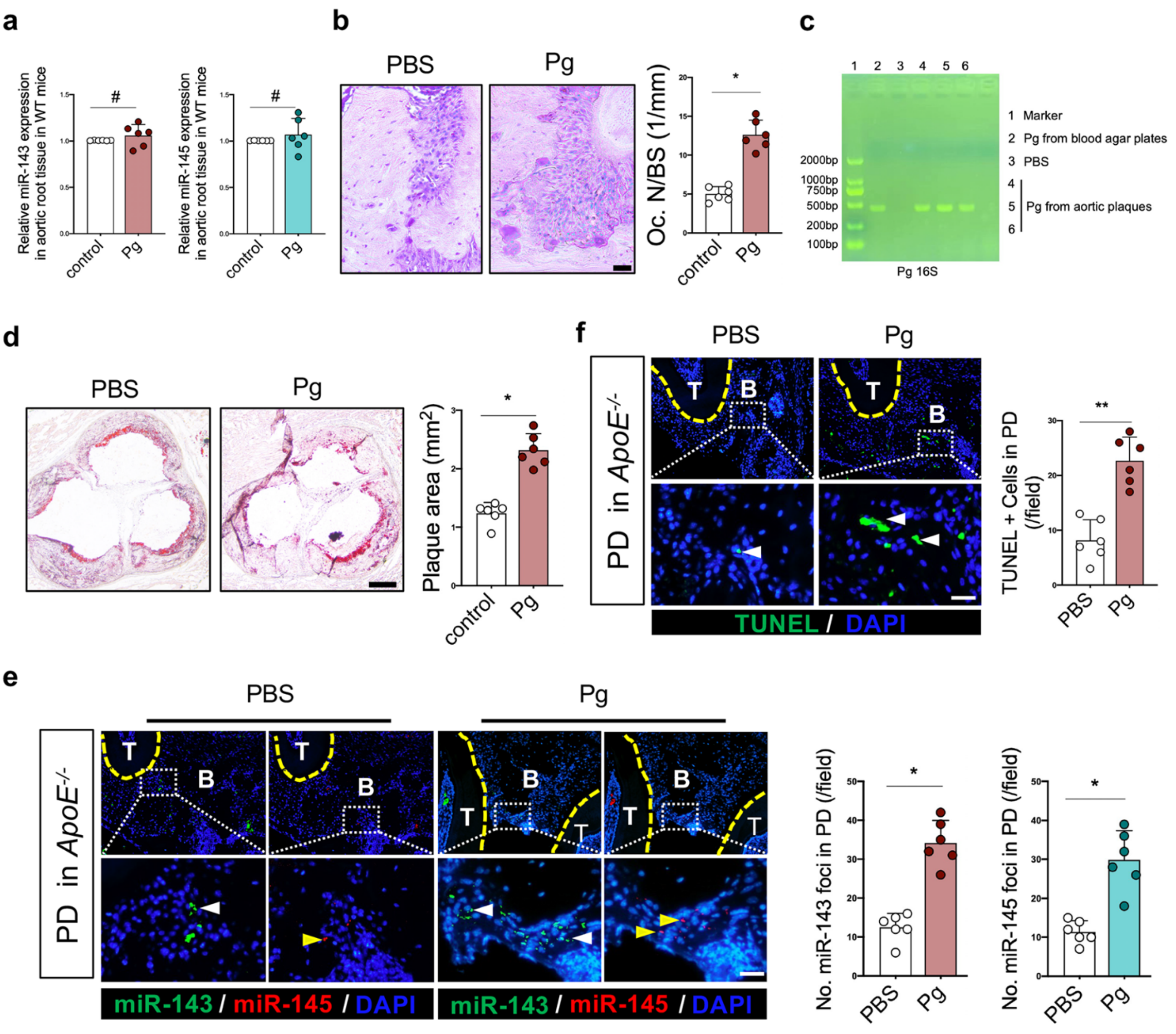

Supplementary Figure 1. Increased miR-143/145 levels and apoptotic cells in *P. gingivalis*-infected *ApoE*$^{-/-}$ mice. (a) qRT-PCR assay showing the miR-143/145 levels in aortic root tissue in the absence or presence of *P. gingivalis* (Pg)-treated wild type (WT) mice. n=6. **(b)** The representative images of osteoclasts on alveolar bone in *ApoE*$^{-/-}$ mice with PBS or *P. gingivalis* exposure after 12 weeks HFD feeding. Right panel showing the quantitative data. Pg: *P. gingivalis*. n=6. Bar: 50 μm. **(c)** Pg from blood agar plated and aortic plaques from *ApoE*$^{-/-}$ mice with PBS or Pg infection were collected for16S rDNA detection by PCR. **(d)** The representative images of Oil Red O staining atherosclerotic lesions of aortic root, with quantitative data at right. n=6. Scale bars: 200 μm. **(e)** The representative images of FISH and **(f)** TUNEL staining showing the miR-143/145 expression and apoptotic cells in periodontitis tissue, respectively. Right panel showing quantitative data. White and yellow arrowhead indicates miR-143 and miR-145, respectively. Yellow dotted line indicates the basement membrane. n=6. Bar: 50 μm. Results are presented as the mean ± S.D by unpaired 2-tailed Student t-tests. *$p < 0.05$; **$p < 0.01$; #$p > 0.05$.

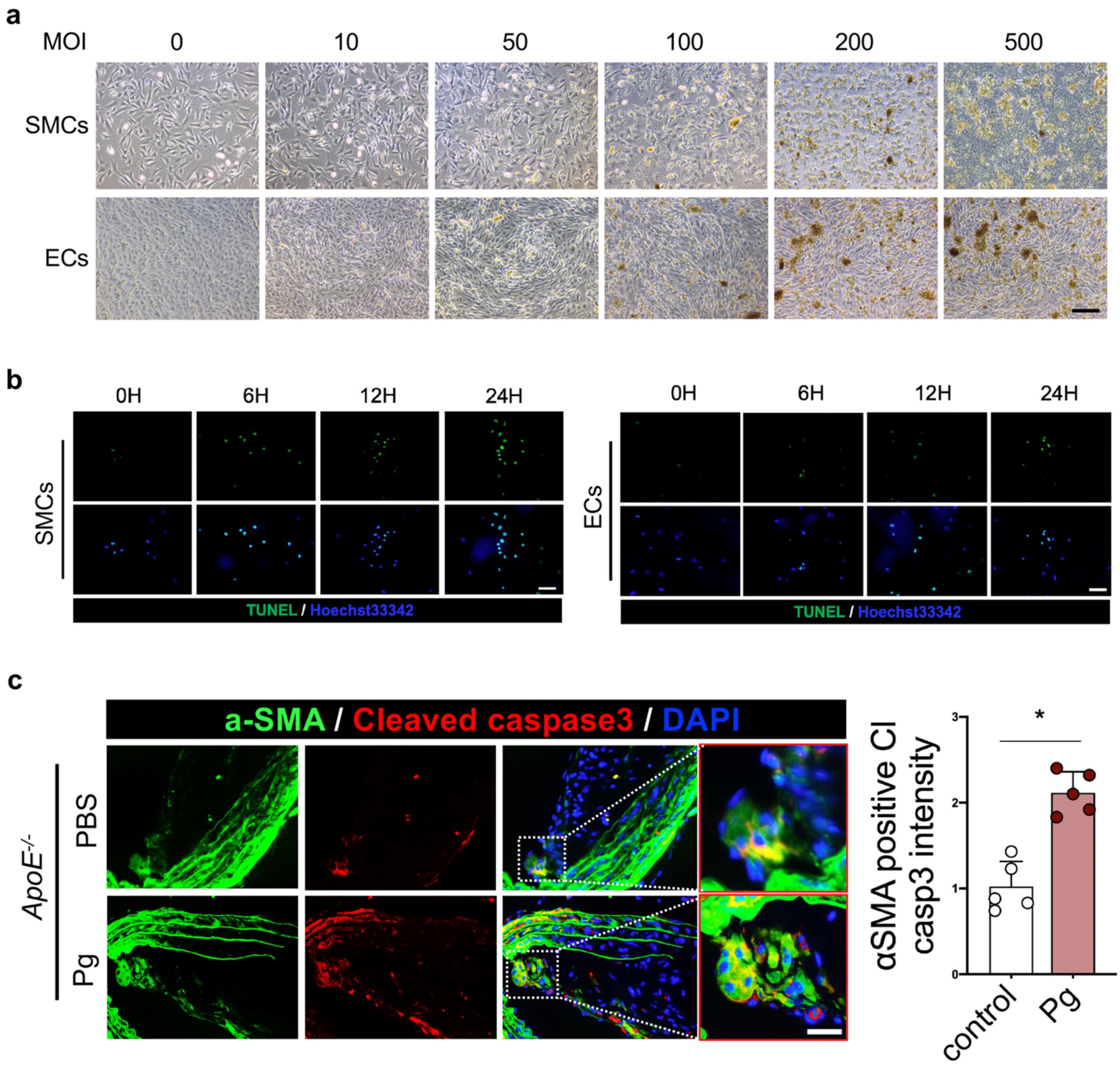

Supplementary Figure 2. SMCs had greater susceptibility to *P. gingivalis*-induced apoptosis than ECs. **(a)** Dead SMCs or ECs were observed under light microscope with different concentration of *P. gingivalis* stimulation. Bar: 200 μm. **(b)** TUNEL staining revealing the apoptotic SMCs or ECs with different concentration of *P. gingivalis* stimulation. Bar: 200 μm. **(c)** Immunofluorescent staining of α-SMA and Cleaved caspase3 revealing the apoptotic SMCs in *ApoE*$^{-/-}$ mice with PBS or *P. gingivalis* treatment, with quantitative data at right. n=6. Bar: 50 μm. Results are presented as the mean ± S.D by unpaired 2-tailed Student t-tests. *$p < 0.05$.

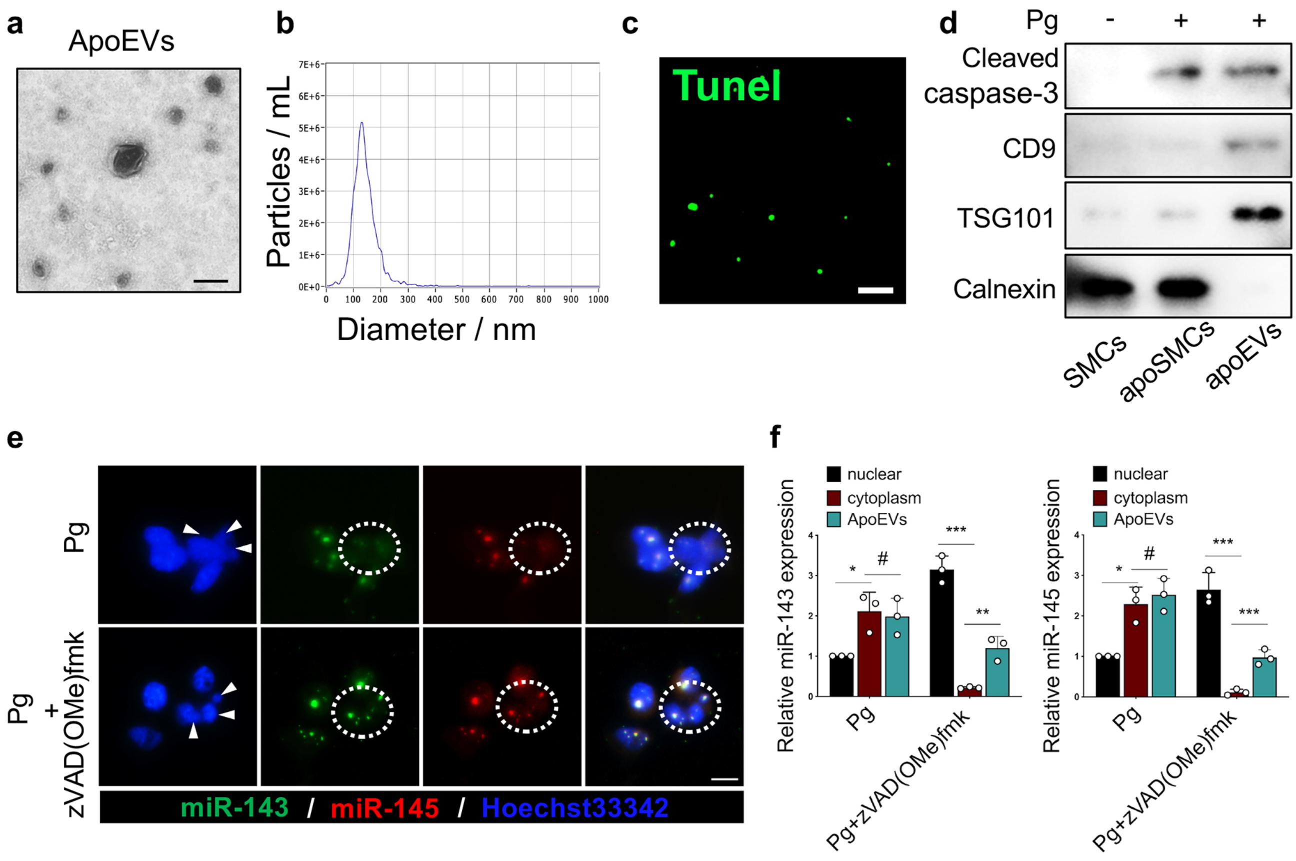

Supplementary Figure 3. Characterization of apoEVs from *P. gingivalis*-induced apoptotic SMCs. (a) A representative image of apoEVs from *P. gingivalis*-induced apoptotic SMCs by transmission electron microscopy (TEM). Scale bars: 100 nm. **(b)** NTA analysis revealing the size distribution of apoEVs. **(c)** Representative microscopy images of apoEVs by TUNEL staining. Scale bars: 20 μm. **(d)** Western blot analysis of SMCs, apoptotic SMCs (apoSMCs), and apoEVs from apoSMCs. Cleaved caspase3 is an apoptosis marker; CD9, TSG101 are extracellular vesicles markers; Calnexin is a cytosolic marker. Pg: *P. gingivalis*. **(e)** Distribution of miR-143 and miR-145 in Pg-infected SMCs after zVAD(OMe)fmk treatment by FISH assay. Scale bars: 20 μm. **(f)** miR-143 and miR-145 levels in nucleus, cytoplasm, apoEVs were examined by qRT-PCR when Pg-infected SMCs were treated with zVAD(OMe)fmk. Results are presented as the mean ± S.D by one-way ANOVA followed with Tukey multiple comparisons tests. *$p < 0.05$; **$p < 0.01$, ***$p < 0.001$; #$p > 0.05$.

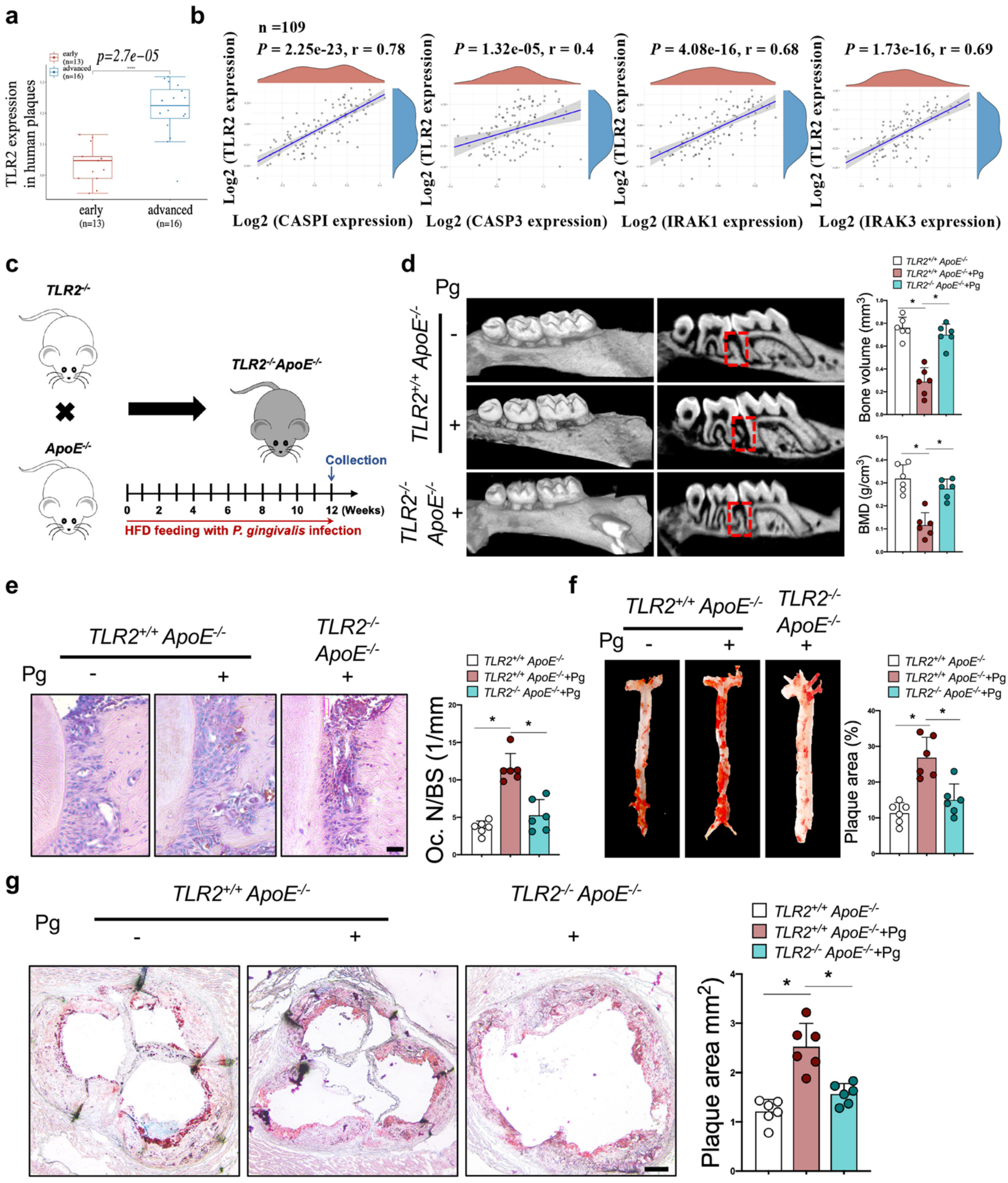

Supplementary Figure 4. The role of TLR2 signaling in *P. gingivalis*-induced SMCs apoptosis, osteoclast activity and plaque formation. (a) Analysis of TLR2 expression in human early (n=13)- and advanced (n=16)- atherosclerotic plaque by RNA-seq from public database. **(b)** Analyzing the correlation between TLR2 and CASPI, CASP3, IRAK1 or IRAK3 in 109 atherosclerotic patients. **(c)** $TLR2^{-/-}ApoE^{-/-}$ mice were constructed for 12 weeks HFD feeding, together with Pg infection. **(d)** micro-CT analysis for alveolar bone between first molar and second molar in $TLR2^{+/+}ApoE^{-/-}$ or $TLR2^{-/-}ApoE^{-/-}$ mice with Pg exposure or not. Right panel showing the quantitative data of bone volume and BMD. Red dotted line indicates ROI. **(e)** TRAP staining for osteoclasts on alveolar bone between first molar and second molar in $TLR2^{+/+}ApoE^{-/-}$ or $TLR2^{-/-}$

ApoE$^{-/-}$ mice with Pg exposure or not after 12 weeks HFD feeding. Right panel showing the quantitative data. n=6. Scale bars: 50 μm. **(f)** The representative images of aorta en face by Oil Red O staining in *TLR2*$^{+/+}$*ApoE*$^{-/-}$ or *TLR2*$^{-/-}$*ApoE*$^{-/-}$ mice after 12 weeks HFD feeding, together with Pg exposure or not. Right panel showing the quantitative data. **(g)** The representative images of aorta root by Oil Red O staining in *TLR2*$^{+/+}$*ApoE*$^{-/-}$ or *TLR2*$^{-/-}$*ApoE*$^{-/-}$ mice with Pg exposure or not after 12 weeks HFD feeding, with quantitative data at right. n=6. Scale bars: 200 μm. Results are presented as the mean ± S.D by one-way ANOVA followed with Tukey multiple comparisons tests. *$p < 0.05$.

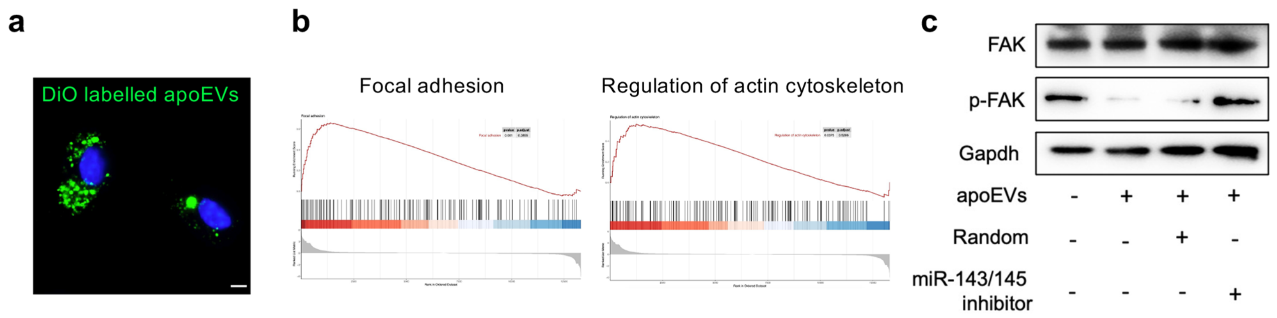

Supplementary Figure 5. ApoEVs-miR-143/145 from apoptotic SMCs regulates focal adhesion pathway in macrophages. (a) ApoEVs labelled with DiO dye were potently incorporated into macrophages. Scale bar: 20 µm. **(b)** Gene set enrichment analysis (GSEA) showed the significant enrichment in focal adhesion and regulation of actin cytoskeleton pathway in miR-143/145-downregulated macrophages. **(c)** Western blot analysis of FAK phosphorylation in the presence of apoEVs or miR-143/145 inhibitor either alone or together.

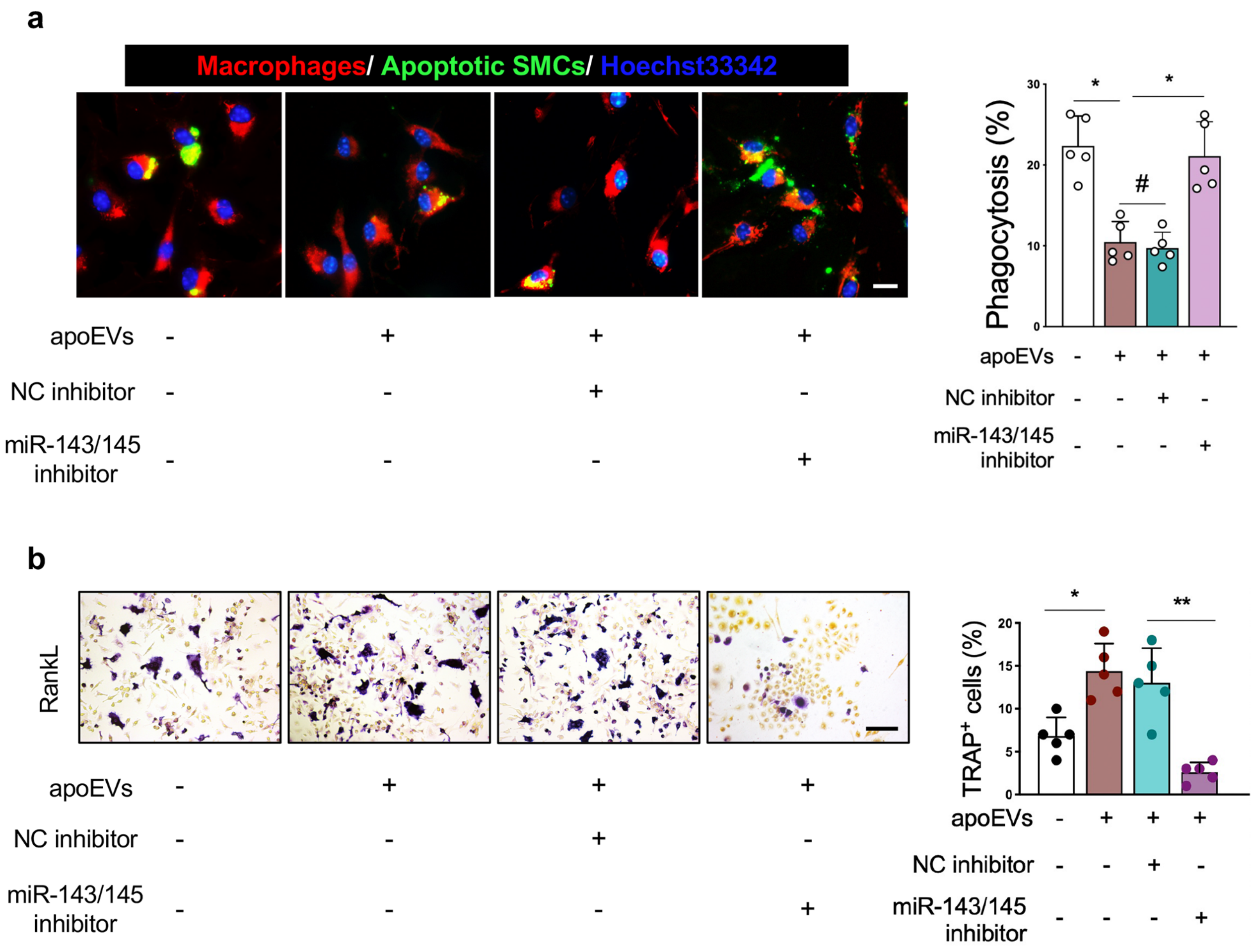

Supplementary Figure 6. ApoEVs-miR-143/145 suppresses efferocytosis but activates osteoclastic differentiation of macrophages. (a) Phagocytosis assay of apoptotic SMCs efferocytosed by macrophages that were treated with apoEVs from *P. gingivalis*-infected SMCs or miR-143/145 inhibitor. Right panel indicating the percentage of efferocytosing macrophages. Scale bars: 20 μm. **(b)** miR-143 or miR-145 mimics either alone or together was co-cultured with RankL-induced macrophages and visualized by TRAP staining. Quantitative measurements of TRAP positive staining cells at right. Scale bars: 100 μm. Results are presented as the mean ± S.D by one-way ANOVA followed with Tukey multiple comparisons tests. *p < 0.05; **p < 0.01; #p > 0.05.

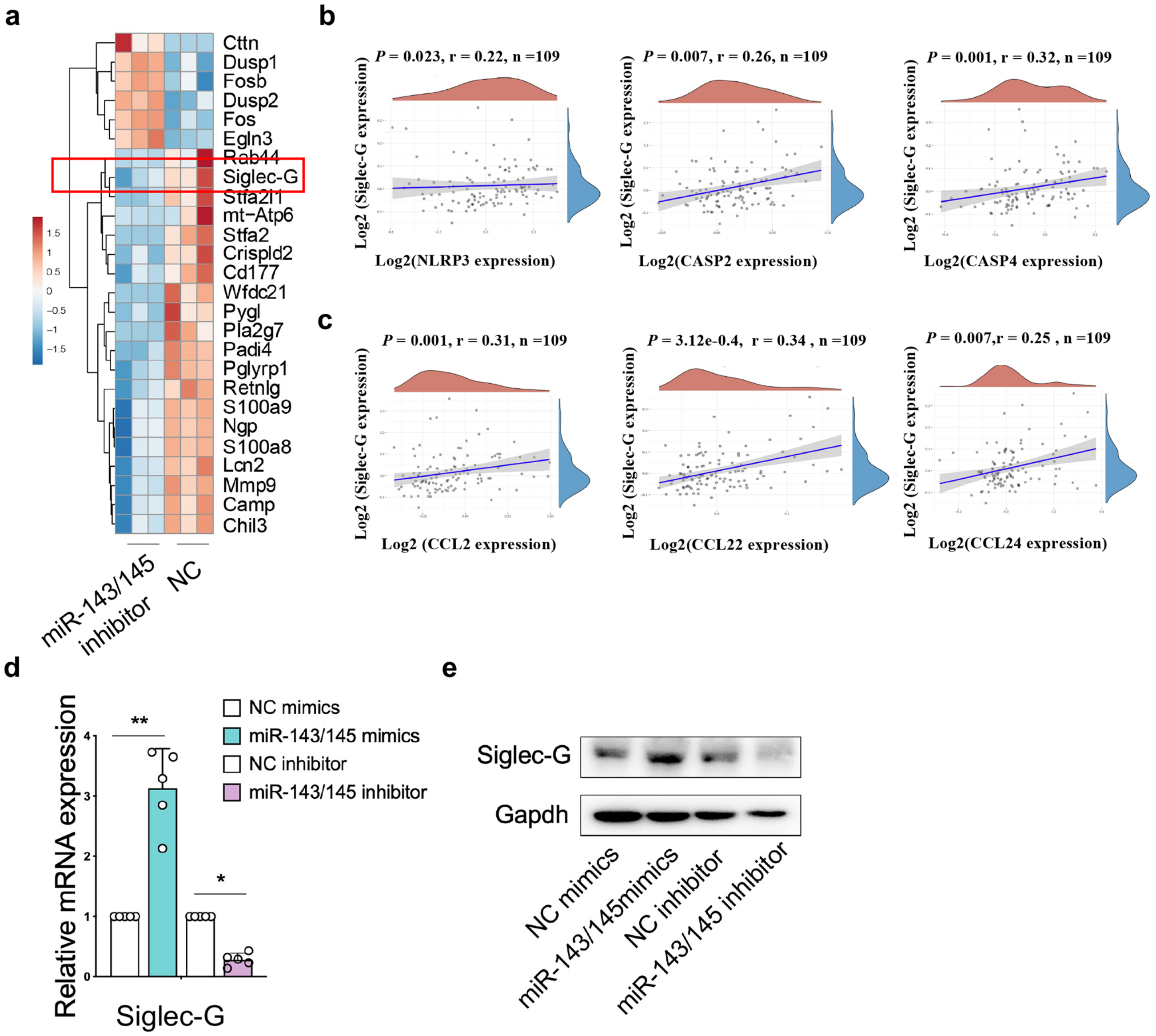

Supplementary Figure 7. Siglec-10 (Siglec-G in mouse) in human atherosclerotic plague by bioinformatics analysis and its expression in macrophages under miR-143/145 control. (a) Heatmap revealing the gene expression profiles of macrophages transfected with negative control (NC) or miR-143/145 inhibitor by RNA-seq. **(b)** Analyzing the correlation between Siglec-10 and NLRP3, CASP2, CASP4 in 109 atherosclerotic patients from public database. **(c)** Correlation between Siglec-10 and CCL2, CCL22, CCL24 in 109 atherosclerotic patients from public database. **(d)** The mRNA and **(e)** protein expression of Siglec-G were increased in miR-143/145-overexpressed macrophages, but decreased with miR-143/145 inhibition. Results are presented as the mean ± S.D by one-way ANOVA followed with Tukey multiple comparisons tests. **p* < 0.05; ***p* < 0.01.

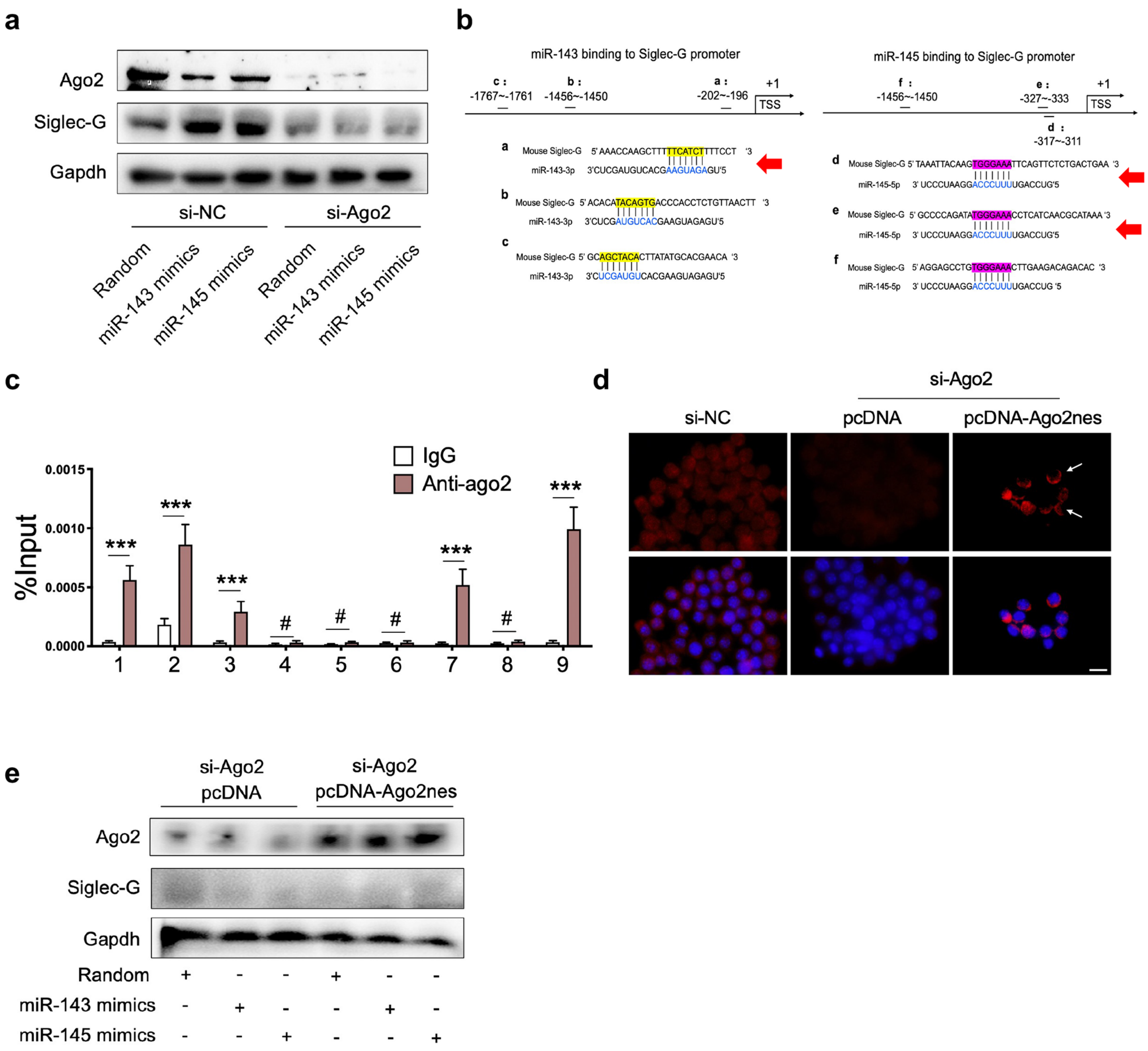

Supplementary Figure 8. miR-143/145 functions in regulation of Siglec-G transcription by cooperating with Ago2. (a) Western blot analysis showed that miR-143 or miR-145 mimics failed to activate Siglec-G expression in the absence of Ago2. **(b)** Sequence alignment of miR-143 and miR-145 binding sites in the promoter of mouse Siglec-G. **(c)** Nine primers were designed to cover the mouse Siglec-G promoter region and used to identify binding sites of Ago2 by ChIP assay. Five putative Ago2-binding sites in Siglec-G promoter region were identified. **(d)** Immunofluorescence staining verified that transfection of pcDNA-Ago2nes re-expressed cytosol Ago2 in Ago2 knockdown macrophages. Arrow indicates the re-expressed cytosol Ago2. **(e)** Effects of miR-143/145 on Siglec-G expression was not rescued with pcDNA-Ago2nes treatment by western blot. Results are presented as the mean ± S.D by one-way ANOVA followed with Tukey multiple comparisons tests. ***$p < 0.001$; #$p > 0.05$.

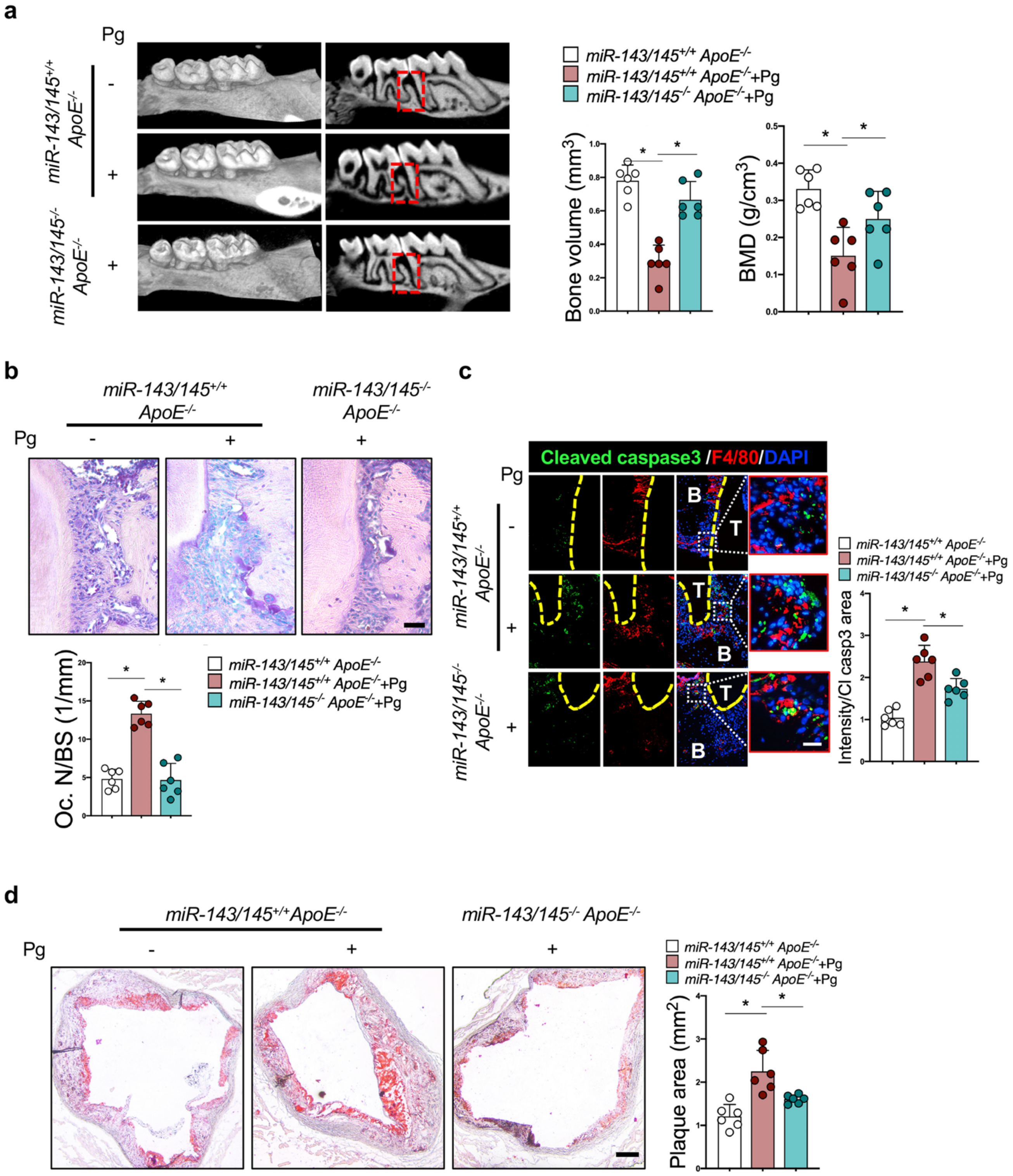

Supplementary Figure 9. The *in vivo* role of miR-143/145 deletion in *ApoE*$^{-/-}$ mice with *P.gingivalis* infection. (a) micro-CT analysis for alveolar bone in *miR-143/145*$^{-/-}$*ApoE*$^{-/-}$ or *miR-143/145*$^{+/+}$*ApoE*$^{-/-}$ mice, with quantitative data of bone volume and BMD at right. Pg: *P. gingivalis*. n=6. **(b)** TRAP staining for osteoclasts in *miR-143/145*$^{-/-}$*ApoE*$^{-/-}$ mice with *P.gingivalis* exposure or not after 12 weeks HFD feeding. Right panel showing the quantitative data. n=6. Scale bars: 50 μm. **(c)** Immunofluorescence staining of Cleaved caspase3 and F4/80 showed the apoptotic cells and macrophages in periodontal tissue, with quantitative data

of apoptotic cells at right. B: bone. T: tooth. Yellow dotted line indicates the tooth root. n=6. Scale bars: 20 μm. **(d)** The representative images of aorta root by Oil Red O staining in *miR-143/145*$^{-/-}$*ApoE*$^{-/-}$ mice with *P.gingivalis* exposure or not after 12 weeks HFD feeding, with quantitative data at right. n=6. Scale bars: 200 μm. Results are presented as the mean ± S.D by one-way ANOVA followed with Tukey multiple comparisons tests. *$p < 0.05$.

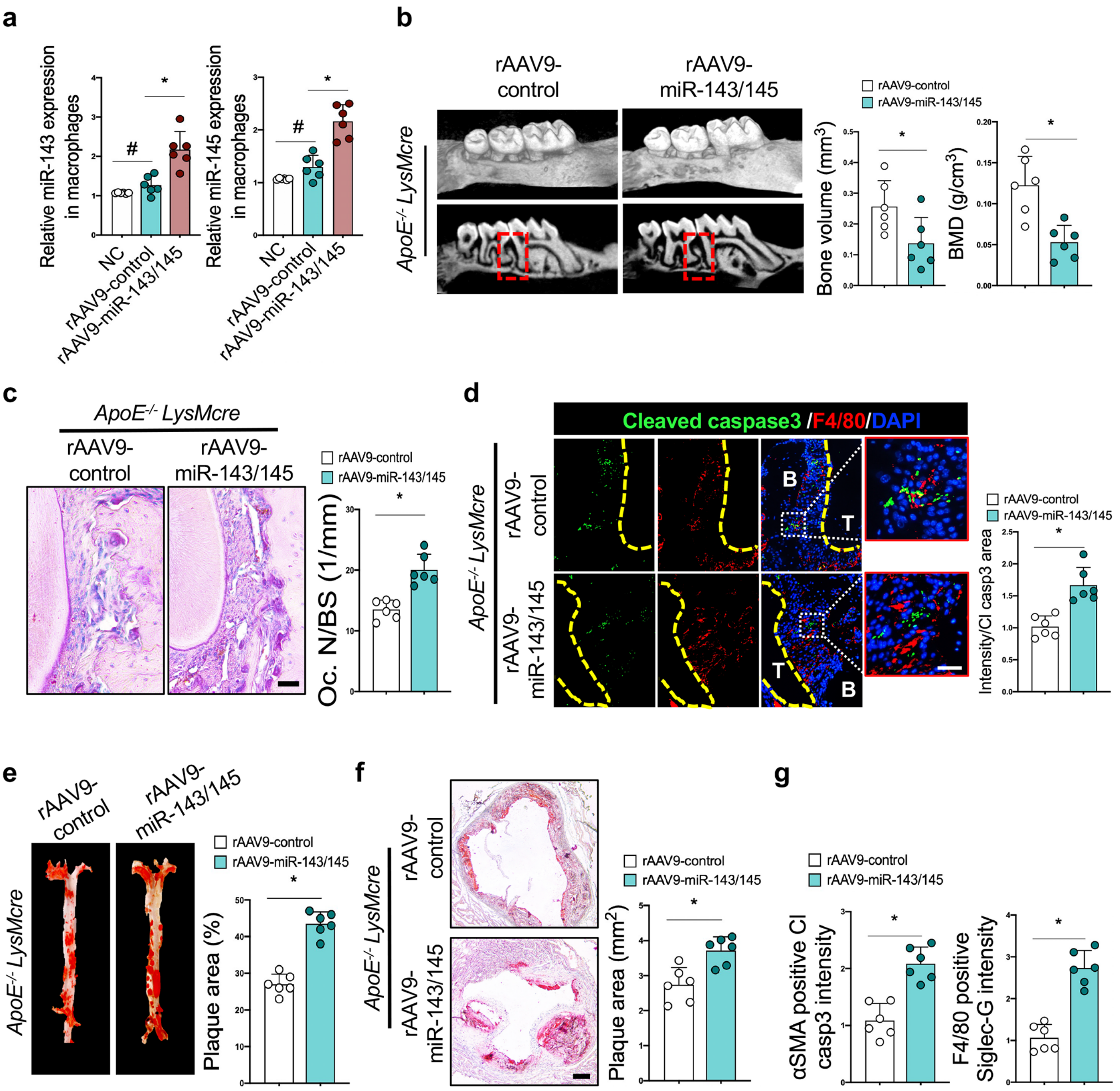

Supplementary Figure 10. Macrophages overexpressing miR-143/145 in *ApoE*$^{-/-}$ mice with *P.gingivalis* infection. (a) After *ApoE*$^{-/-}$*LysMcre* mice were intravenously injected with rAAV9-control or rAAV9-miR-143/145 vector, miR-143 and miR-145 levels in macrophages were examined by qRT-PCR. **(b)** micro-CT analysis for alveolar bone in rAAV9-control or rAAV9-miR-143/145 injected *ApoE*$^{-/-}$*LysMcre* mice with Pg infection or not after 12 weeks HFD feeding. n=6. **(c)** TRAP staining for osteoclasts in *ApoE*$^{-/-}$*LysMcre* mice with Pg infection or not for 12 weeks HFD feeding and intravenously injected with rAAV9-control or rAAV9-miR-143/145 vector. Right panel showed the quantitative data. n=6. Scale bars: 50 µm. **(d)** Immunofluorescence staining showed the apoptotic cells and macrophages in periodontal tissue, with quantitative data at right. B: bone. T: tooth. Yellow dotted line indicates the tooth root. n=6. Scale bars: 20 µm. **(e)** The representative images of atherosclerotic lesions of aorta en face, with quantitative data of plaque area at right. n=6. **(f)** The representative images of aorta root by Oil Red O staining, with quantitative data at

right. n=6. Scale bars: 200 μm. **(g)** Quantitative analysis of apoptotic SMCs and Siglec-G$^+$ macrophages in serial plaque sections from *miR-143/145*$^{-/-}$*ApoE*$^{-/-}$ or *miR-143/145*$^{+/+}$*ApoE*$^{-/-}$ mice. Results are presented as the mean ± S.D by one-way ANOVA followed with Tukey multiple comparisons tests or unpaired 2-tailed Student t-tests. *$p < 0.05$; #$p > 0.05$.

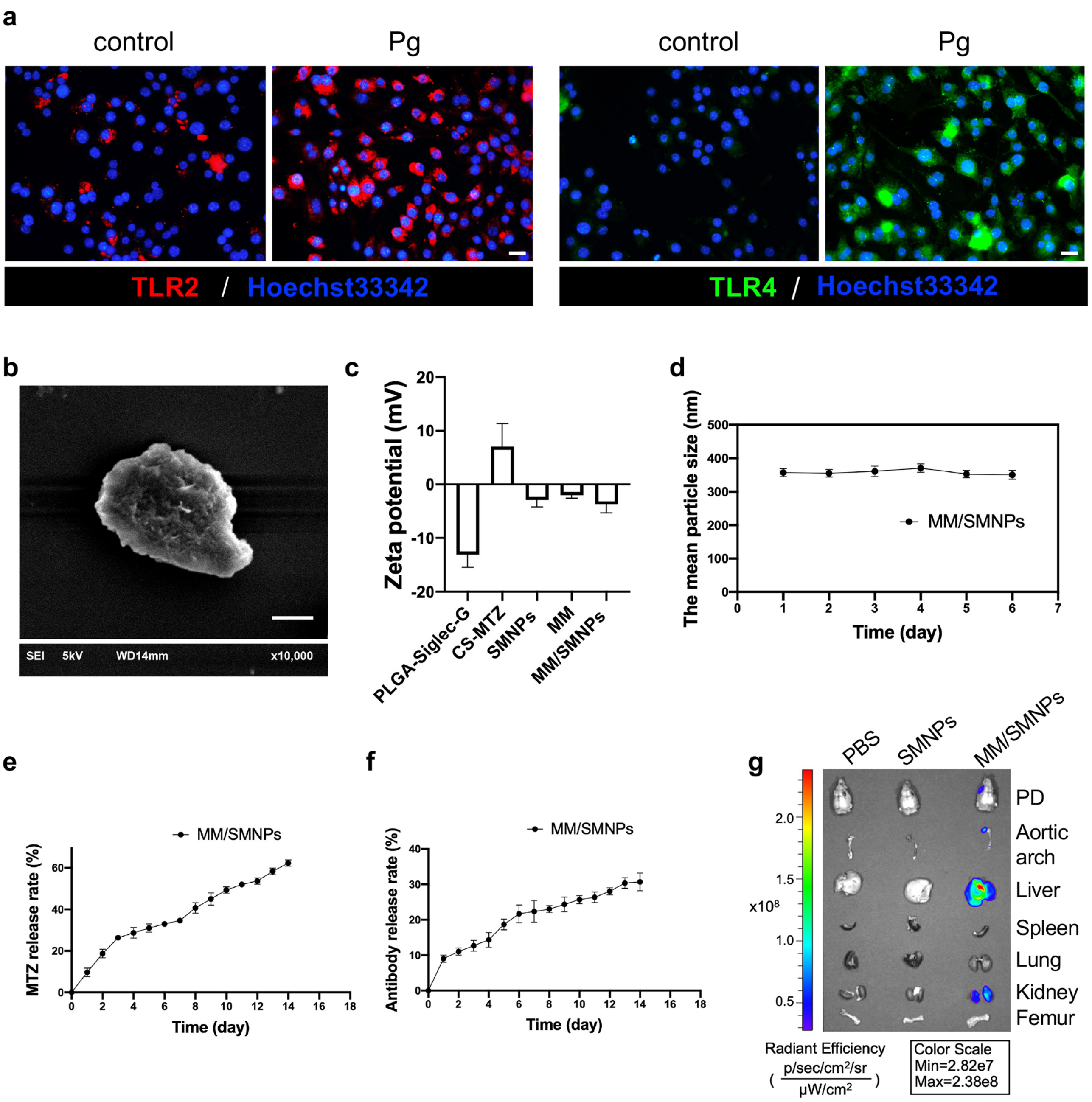

Supplementary Figure 11. Characterization of *P.gingivalis* pretreated macrophages and release profile of MM/SMNPs nanoparticles. (a) Immunofluorescence staining of TLR2 and TLR4 in macrophages. Hoechst33342 indicated nuclei. **(b)** SEM images of MM/SMNPs. Scale bar: 100 nm. **(c)** The zeta potential was detected in the process of MM/SMNPs preparation. **(d)** Stability of MM/SMNPs in PBS with a span of 6 days. **(e)** Present of metronidazole (MTZ) release was calculated with a span of 14 days by UV−vis spectra analysis. **(f)** Present of anti-Siglec-G antibody release was calculated with a span of 14 days by BSA protein detection. **(g)** After intravenously injection with ICG loaded MM/SMNPs for 24 hours, the fluorescence signal in the heart, liver, spleen, lung, kidney and femur was monitored by IVIS optical imaging. Y-axis indicates radiant efficiency (p/s/cm2/sr/μW/cm2).

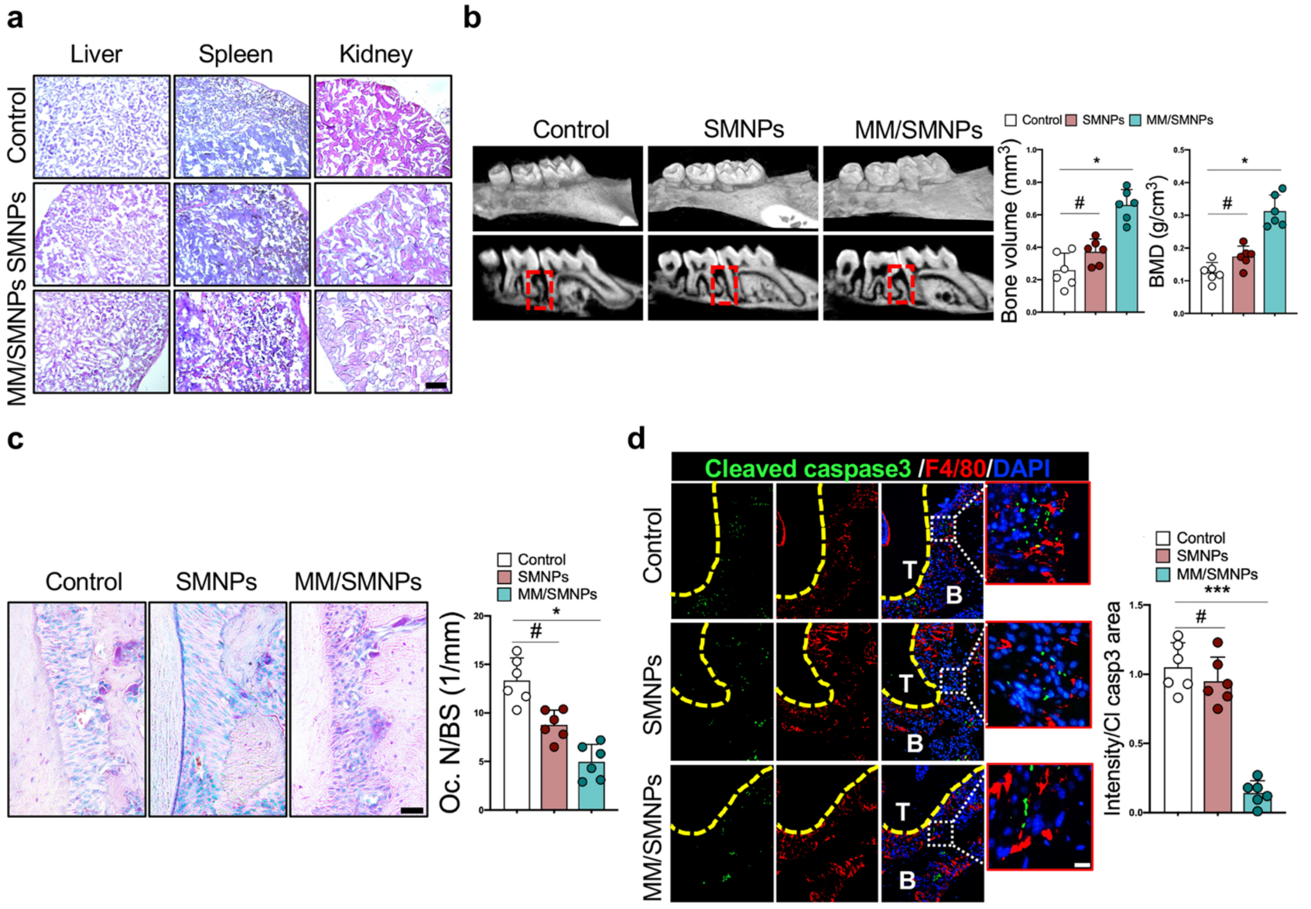

Supplementary Figure 12. Therapeutic effect of MM/SMNPs nanoparticles on periodontitis in *ApoE*$^{-/-}$ mice. (a) H&E staining of liver, spleen, and kidney from control, SMNPs-, and SMNPs and MM/SMNPs-treated *ApoE*$^{-/-}$ mice. Scale bars: 200 µm. **(b)** Alveolar bone in *ApoE*$^{-/-}$ mice treated with PBS (control), SMNPs and MM/SMNPs was assessed by 3D reconstruction and relative quantification of micro-CT. n=6. **(c)** TRAP staining for osteoclasts in *ApoE*$^{-/-}$ mice with control, SMNPs and MM/SMNPs treatment. Right panel showed the quantitative data. n=6. Scale bars: 50 µm. **(d)** Immunofluorescence staining showed the apoptotic cells and macrophages in periodontal tissue, with quantitative data at right. B: bone. T: tooth. Yellow dotted line indicates the tooth root. n=6. Scale bars: 20 µm. Results are presented as the mean ± S.D by one-way ANOVA followed with Tukey multiple comparisons tests. $*p < 0.05$; $***p < 0.001$; $\#p > 0.05$.
